# PM_2.5_ exposure disparities persist despite strict vehicle emissions controls in California

**DOI:** 10.1126/sciadv.adn8544

**Published:** 2024-09-11

**Authors:** Libby H. Koolik, Álvaro Alvarado, Amy Budahn, Laurel Plummer, Julian D. Marshall, Joshua S. Apte

**Affiliations:** ^1^ Department of Civil and Environmental Engineering, University of California, Berkeley, Berkeley CA 94720, USA.; ^2^ California Office of Environmental Health Hazard Assessment, Sacramento, CA 95814, USA.; ^3^ Department of Civil and Environmental Engineering, University of Washington, Seattle, WA 98195, USA.; ^4^ School of Public Health, University of California, Berkeley, Berkeley, CA 94704, USA.

## Abstract

As policymakers increasingly focus on environmental justice, a key question is whether emissions reductions aimed at addressing air quality or climate change can also ameliorate persistent air pollution exposure disparities. We examine evidence from California’s aggressive vehicle emissions control policy from 2000 to 2019. We find a 65% reduction in modeled statewide average exposure to PM_2.5_ from on-road vehicles, yet for people of color and overburdened community residents, relative exposure disparities increased. Light-duty vehicle emissions are the main driver of the exposure and exposure disparity, although smaller contributions from heavy-duty vehicles especially affect some overburdened groups. Our findings suggest that a continued trend of emissions reductions will likely reduce concentrations and absolute disparity but may not reduce relative disparities without greater attention to the systemic factors leading to this disparity.

## Teaser

Decades of California’s strict exhaust emissions controls reduced PM_2.5_ exposure but not relative racial-ethnic disparity.

## INTRODUCTION

Despite decades of progress in improving ambient air pollution in the United States, people of color still bear a disparate burden of air pollution (*1*–*12*). Within California, research has quantified and characterized these exposure disparities using both measurements and models (*13*–*18*). Solutions to this persistent inequality are increasingly a focus for academic research and environmental policy at the federal, state, and local levels (*9*, *17*–*19*). A growing body of research investigates how air quality policies might contribute to a “triple win” that simultaneously achieves meaningful benefits by reducing population-wide exposures, mitigating greenhouse gas emissions, and reducing exposure disparities and extreme exposures (*20*, *21*). Here, we use a modeling framework to explore how multi-decade emissions reductions shaped by air quality and climate policies have affected environmental justice (EJ) outcomes, using California’s aggressive on-road mobile source strategy as a case study. In this work, we focus on exposure disparities, which can be distinct from disparities in health outcomes (*22*–*27*).

Recent research on how to reduce air pollution exposure disparities in the United States presents two conflicting approaches (*9*, *19*–*21*, *28*). The first approach starts from the recognition that many major emitting sectors lead to disparate exposures for people of color (*2*). Thus, focusing on emissions reductions for sectors that especially affect people of color could have EJ co-benefits (*21*, *29*–*32*). This approach mirrors the policy structure in the United States and elsewhere, where environmental regulations are targeted to individual economic sectors (e.g., vehicles, industries, and power plants) and tailored to relevant technology and infrastructure. The second body of research suggests that sector-oriented policies may not be effective in addressing relative disparities in air pollution. For example, optimization modeling found that aggressive nationwide emissions reductions targeting economic sectors with higher-than-average disparity impact would not eliminate racial-ethnic fine particulate matter (PM_2.5_) exposure disparities without nearly eliminating emissions (*9*). In contrast, a location-specific approach—i.e., emissions reductions by location rather than by economic sector—rapidly eliminated disparities. Building upon this finding, two recent studies (*20*, *21*) simulated climate policies with substantial abatement of PM_2.5_ and its precursors across most US economic sectors and found modest potential reductions in disparities. They too reported that “location-specific” policies that target emissions reductions in all sectors within specific overburdened geographies may have a high potential to address relative exposure disparities even with small emissions changes (*9*, *19*). To complement prospective studies, which consider ways to reduce future exposure disparity, we examine the disparity impacts of historical emissions trajectories. We focus on the transportation sector, which is often highlighted as having a high potential to reduce exposure disparities. Historically, racist urban planning and infrastructure decisions (e.g., redlining and freeway siting) have concentrated vehicle emissions in communities of color (*2*, *4*, *7*, *13*, *29*). Furthermore, people who are exposed to the highest levels of traffic-related air pollution often are not the communities who drive the most (*30*–*32*). Hence, a recent study found that emissions controls for the transportation sector have the greatest potential to mitigate racial-ethnic inequality in US air pollution (*21*). Simultaneously, the transportation sector is a priority area for regulatory agencies and EJ-oriented community groups; emissions reductions from these sources could potentially reduce exposure disparities, human health impacts, and greenhouse gas emissions (*33*).

For nearly 60 years, California led the United States in reducing on-road vehicle emissions. Because California’s motor vehicle emission regulation preceded the Clean Air Act of 1970, California is delegated the authority to set vehicle emissions standards more stringently than the federal equivalent (*34*–*36*). In the present analysis, we model exposure concentrations for the years 2000 through 2019, during which California’s regulatory agencies pursued an aggressive and interlinked suite of multi-pollutant policies to reduce emissions across the entire on-road vehicle fleet (*36*). Examples include requiring cleaner fuels and technological advancements (e.g., hybrid drivetrain, alternative fuel and propulsion technologies, and advanced emissions controls) specific to light-duty, medium-duty, and heavy-duty vehicle classes (LDV, MDV, and HDV, respectively).

The suite of regulations that comprise California’s mobile source strategy has resulted in large aggregate reductions of emissions of multiple pollutants from diverse fleets that make up the state’s on- and off-road vehicles (*37*). Here, we examine how changes in on-road vehicle emissions from 2000 to 2019 have affected exposure to PM_2.5_. Over this time period, on-road emissions have been shaped by several aggressive state regulations targeting specific vehicle fleets, including California Air Resources Board’s (CARB) Light-Duty Vehicle Emissions Standards, Advanced Clean Cars, and the Truck and Bus Regulation (*38*). Despite statewide and fleetwide on-road vehicle miles traveled increasing ~24%—from 292 billion (2000) to 364 billion (2019)—emissions of the four species that principally drive population-weighted PM_2.5_ exposures from on-road vehicles have decreased. Regulatory emissions data indicate reductions of ~70% for primary PM_2.5_, nitrogen oxides (NO*_x_*), and volatile organic compounds (VOC), while ammonia (NH_3_) decreased by ~15% (fig. S1) (*38*). Notably, non-exhaust primary PM_2.5_ emissions (e.g., brake and tire wear) have increased by ~20% over this time period, causing the relative non-exhaust share of primary PM_2.5_ to increase substantially (14 to 50% from 2000 to 2019) (*39*). Diverse measurement and observational datasets (see the Supplementary Materials) corroborate overall declining emissions of PM, NO*_x_*, VOC, NH_3_, and other key traffic-related air pollutants (TRAPs) (*40*–*50*). Considering all species that contribute to total PM_2.5_, California’s on-road emissions reductions outpaced the national aggregate, especially for NO*_x_* and VOC (*51*).

On-road vehicle emissions are anticipated to continue to decline in California in response to major new regulations: Advanced Clean Cars II (starting in 2035, requires all new passenger cars, trucks, and SUVs sold in California to be zero-emission vehicles) and Advanced Clean Fleets (starting in 2045, all trucks that drive in California must use zero-emissions technology). A few recent studies have projected the air pollution and equity impacts of vehicle electrification in California and found limited equity benefits. In this paper, we build on a much smaller body of work (*14*, *52*) to focus retrospectively on the equity impacts of past changes in vehicle emissions over two recent decades to inform future policy.

We investigate whether the combined impacts of the ensemble of mobile source strategies have contributed to a reduction in PM_2.5_ exposure disparities. Exposure disparities are multifaceted; we quantify them along several axes described below. Our analysis also considers two specific features (vehicle type and spatial scale) that are central to current regulatory design. We conclude with implications from this California-focused retrospective analysis for future EJ-focused policy for the United States.

We developed and used an open-source analysis method based on atmospheric simulations from the Intervention Model for Air Pollution (InMAP; see Materials and Methods) to model total PM_2.5_ concentrations resulting from emissions of PM_2.5_, NO*_x_*, VOC, NH_3_, and sulfur oxides (SO_x_) emitted by California’s on-road mobile source sector from 2000 to 2019. Estimates of on-road mobile emissions are from CARB’s EMission FACtor regulatory model (EMFAC v2021 with MPOv11), which has been approved by the US EPA (*53*). EMFAC represents CARB’s best estimate of on-road emissions; it incorporates detailed administrative and observational data pertaining to fleet composition, emissions performance, and spatiotemporal activity patterns (*38*). Variably sized gridded PM_2.5_ concentrations (1 to 48 km, higher resolution in greater population density locations) are combined with tract-level 2010 census population data to estimate exposure disparities among demographic groups (*15*). We disaggregate mobile source impacts into four vehicle types: LDV, MDV, HDV, and all other vehicles (e.g., buses, motorcycles, and motorhomes; table S1).

In the United States and California, air pollution exposure disparities tend to be larger by race-ethnicity than by other socioeconomic and demographic indicators (e.g., income, education, and urbanicity) due in large part to the historical racism and racist practices (e.g., housing discrimination, redlining, and highway relocation) that segregated cities and placed high-pollution sources near communities of color (*2*–*4*, *10*, *11*, *54*). Accordingly, we focus our analyses on racial-ethnic disparities. In addition, we consider two statutory geographic designations (AB617 and SB535) of cumulative impacts that California uses for prioritizing EJ (fig. S2) (*55*, *56*). Although these geographies have only recently been established (and thus past policy may or may not have explicitly targeted these places), we focus on them here because they are examples of location-specific policies that target emissions reductions in overburdened communities. Through the Community Air Protection Program (AB617), California has designated specific communities (2.7 million people, year 2010; 8.1% of the state’s population) for priority in community-based air pollution monitoring and emissions reduction plans (*55*). A second policy, SB535 (10.2 million people, 30.0% of the state’s population), focuses on targeting financial investments toward people living in “disadvantaged communities,” identified using several environmental, socioeconomic, and public health indicators for each US census tract in California (*56*, *57*).

From here onward, we use the term “overburdened communities” to refer specifically to the areas designated as AB617 or SB535 communities and refer to the people who live in these areas as “residents of overburdened communities.” The demographic makeup of residents of overburdened communities has a higher proportion of people of color (all groups except for non-Hispanic white Californians) than the statewide population (people of color: 92.9% in AB617 communities, 82.9% in SB535 communities; table S2). We also specifically consider exposure and disparities experienced by individual racial-ethnic groups (e.g., Hispanic Californians).

## RESULTS AND DISCUSSION

### Statewide trends in overall exposure and relative exposure disparity

**Fig. 1.**
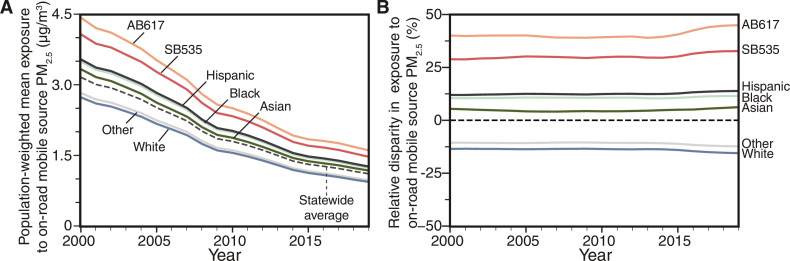
On-road mobile source PM_2.5_ exposure and relative disparity in exposure for each demographic group. Statewide population-weighted mean PM_2.5_ exposure concentrations (**A**) and relative disparity in exposure (**B**) attributable to on-road mobile sources for the four largest racial-ethnic groups and two policy-relevant environmental justice areas in California. In each year, relative exposure disparities (B) for each racial-ethnic group are computed in reference to the statewide average PM_2.5_ concentration attributable to on-road mobile sources. Concentrations in overburdened communities designated under California’s Community Air Protection Program (AB617, ~10% of state population) and as SB535 Disadvantaged Communities (~25% of state population) substantially exceed those experienced on average for the most exposed racial-ethnic group, Hispanic Californians. Crucially, despite greater than 50% reductions in mobile source population-weighted mean PM_2.5_ for all groups (A), relative racial-ethnic disparities increased for Hispanic, Black, and Asian Californians, as well as residents of overburdened communities. Here and elsewhere, the “Hispanic” population reflects Californians of any racial group identifying on the US census as Hispanic, while all other groupings exclude Californians identifying as Hispanic.

California’s mobile source policy has succeeded in its overall goal of reducing PM_2.5_ exposures (Fig. 1A). We find that the modeled statewide population-weighted mean (PWM) PM_2.5_ exposure concentration attributable to on-road vehicles decreased from approximately 3.2 to 1.1 μg/m^3^ from 2000 to 2019, a ~65% decrease (i.e., nearly a factor of 3) in exposure on average for all Californians. This reduction in PM_2.5_ exposure from on-road vehicles outpaced the overall statewide improvement in ambient air quality (fig. S3). For context, multiple independent estimates of total PWM PM_2.5_ from all sources in California show an approximate ~40% decrease from 15 to 9 μg/m^3^ from 2000 to 2019 (*58*–*60*).

We evaluate PWM PM_2.5_ exposure from on-road mobile sources for racial-ethnic groups and residents of overburdened communities (Fig. 1A). Our modeled estimate of PM_2.5_ declined for all groups, and the ordering of exposures by group is generally consistent over time. Among all racial-ethnic groups, Hispanic Californians experienced the highest exposure for all years, with PWM PM_2.5_ exposure concentrations of approximately 3.5 and 1.3 μg/m^3^ in 2000 and 2019, respectively. Black Californians experienced the next highest PWM exposure concentration (3.5, 1.2 μg/m^3^ in 2000 and 2019, respectively), followed by Asian Californians (3.3, 1.2 μg/m^3^). Of the four racial-ethnic groups in Fig. 1A, white Californians were exposed to the lowest PWM PM_2.5_ concentrations: approximately 2.7 and 0.9 μg/m^3^ from 2000 and 2019, respectively. Residents of overburdened communities were exposed to substantially higher PWM concentrations of PM_2.5_ from on-road mobile sources (AB617 residents: 4.4, 1.6 μg/m^3^ in 2000 and 2019; SB535 residents: 4.1, 1.5 μg/m^3^ in 2000 and 2019) than the PWM for any racial-ethnic group shown in Fig. 1A.

For each demographic group, we compute exposure disparity as the absolute (in micrograms per cubic meter) and relative (percent) difference between the average modeled concentration experienced by a group versus the overall state population (Fig. 1B and table S3; see also Materials and Methods). In this work, we discuss exposure disparities in both absolute and relative terms. Both metrics provide useful insights into exposure inequality. Because increases in PM_2.5_ concentration have a causal relationship with increases in adverse health outcomes, absolute differences between groups of people must be minimized to the extent possible. However, systemic inequality in terms of relative exposure disparity can persist even if the most overburdened areas receive the largest reductions in exposure in absolute terms if those reductions are not also the largest in percentage terms. Crucially, our analyses focus exclusively on PM_2.5_ exposure disparities attributable to on-road vehicles. Most other major emitting sectors in California also disparately expose residents of overburdened communities and people of color to PM_2.5_ (*2*, *15*). Likewise, exposures to other air pollutants are also unequally distributed (*4*, *7*, *10*). Accordingly, when we find that disparities persist, they persist in a larger story of environmental inequity in California.

Reflecting the nearly parallel exposure concentration traces over time evident in Fig. 1A, relative disparities in PM_2.5_ exposure from on-road mobile sources (Fig. 1B) were notably persistent, increasing slightly over this time period. The relative disparity in exposure to on-road mobile sources for Hispanic Californians increased slightly from 12.0 (the year 2000) to 13.9% (the year 2019), while the relative disparity in exposure for white Californians decreased slightly from −13.5 to −15.5%. Thus, the overall relative difference between the most and least exposed race-ethnicity increased from 30 to 35%. Given expected model uncertainties, these incremental changes may not necessarily represent evidence of a trend that is distinguishable from approximately constant relative disparity. Likewise, relative disparities for Black and Asian Californians also persisted (respectively 10.5 to 11.5% and 5.4 to 6.2% over this period). Exposure disparities by race-ethnicity are larger than by income (fig. S4). Notably, we find persistent disparities in exposure to both primary and secondary PM_2.5_ from vehicle emissions. Relative disparities in exposure to primary PM_2.5_ components (18.6% for Hispanic Californians) were larger than disparities in exposure to secondary PM_2.5_ (11.1% for Hispanic Californians).

Absolute and relative exposure disparities in overburdened communities are even larger. For example, the relative disparity in exposure (i.e., relative to the overall population average) for on-road mobile source PM_2.5_ is more than three times as large for AB617 communities as for the most-exposed racial-ethnic group, increasing somewhat from 40 (the year 2000) to 45% (the year 2019). Stratifying by CalEnviroScreen score (which is used in part to identify SB535 communities), we find even larger relative disparities (fig. S4).

Disparities in exposure for those living at the extreme ends of the concentration distribution are also relevant for understanding environmental injustice. We estimated population-weighted distributions of exposure by race-ethnicity for each vehicle class (figs. S5 and S6). In general, changes in exposure at the upper percentiles (i.e., 75th and 90th) are consistent with changes in exposure at the PWM and consistent across time. Considering the disparity in exposure at the 75th and 90th percentiles relative to the statewide mean, we find large and increasing relative disparities (e.g., 90th percentile exposure for Hispanic Californians increased from 104 to 118% higher than statewide PWM from 2000 to 2019).

**Fig. 2.**
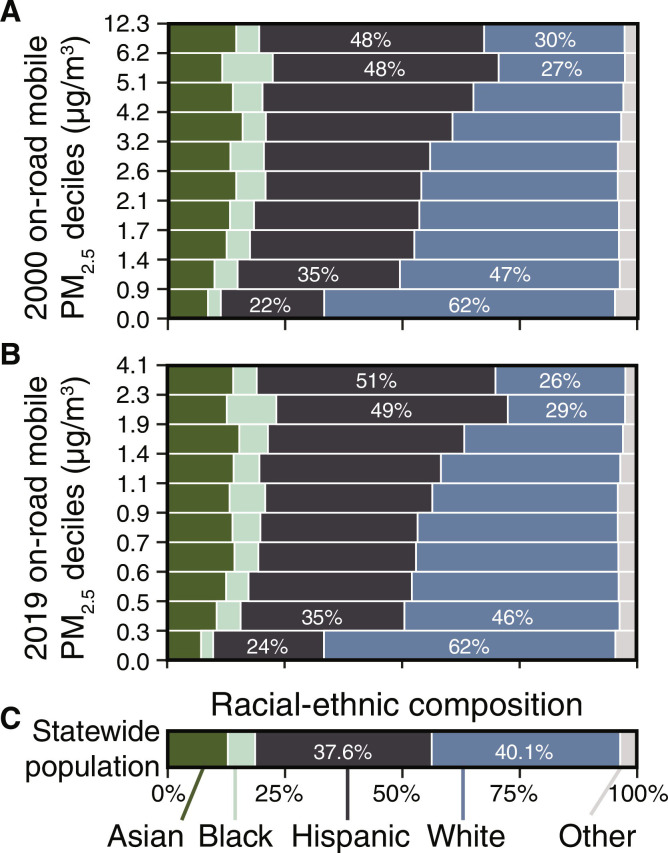
Racial-ethnic population distribution by exposure decile. Differences in the racial-ethnic composition of the California population exposed to each decile of the distribution of PM_2.5_ attributable to on-road mobile sources in (**A**) 2000 and (**B**) 2019. The statewide population is binned into 10 groups of equal population of PM_2.5_ exposure attributable to the full vehicle fleet. At all years in our assessment, Hispanic Californians are strongly overrepresented among the highest PM_2.5_ exposure deciles (and underrepresented in the lowest exposure deciles). The opposite pattern holds for white Californians. Data are plotted for individual vehicle types and the analysis midpoint year (2010) in the Supplementary Materials.

We also evaluated the degree to which the California populations who experience the highest overall exposure to PM_2.5_ from on-road vehicles are disproportionately composed of people of color, and how this pattern has evolved over time. To do so, we binned the California population by decile of modeled exposure to PM_2.5_ and then compared the racial-ethnic composition of each decile in 2000 and 2019 (Fig. 2 and midpoint result in fig. S7). From 2000 to 2019, Hispanic Californians are overrepresented at the highest exposure deciles. While the California state population is 37.6% Hispanic, the highest decile of exposure for emissions in 2000 and 2019 consists of 47.9 and 50.8% Hispanic people, respectively. Similarly, white Californians, who comprise 40.1% of the population, are overrepresented among the populations with the lowest exposures (62.0% of the lowest-exposure deciles in 2000 and 2019) and are underrepresented in the highest-exposure decile [29.9% (2000) and 27.7% (2019)]. In fig. S8, we examine the racial-ethnic composition of the population across the full distribution of absolute and percentage changes in PM_2.5_ exposure from on-road vehicles. While the grid cells with the largest absolute reduction in concentration consist of more people of color than the statewide average, there are only small demographic differences in the percentage change in exposure. This result arises in large part because the geographies with the largest absolute reductions in PM_2.5_ exposure from on-road mobile sources started with the highest initial levels of exposure in 2000.

### Differences in contributions to exposure disparity by fleet type

Because California’s vehicle emissions control policies generally differentiate by vehicle type, we disaggregate our analyses of emissions, exposures, and disparities by vehicle type based on the official EMFAC2021 documentation (table S1) (*38*). We model the disparities and additive contributions of each vehicle fleet type at the state level for the most exposed racial-ethnic group, Hispanic Californians, to identify which vehicle types have an especially influential role in their exposures and disparities.

**Fig. 3.**
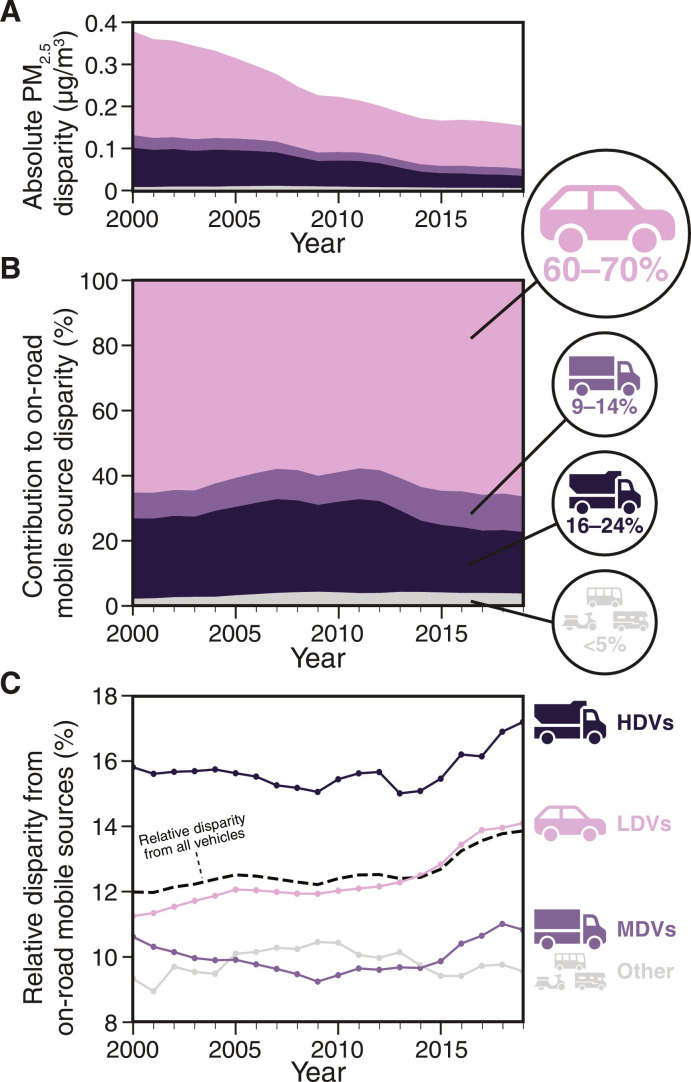
Contributions to disparity in exposure to mobile-source PM_2.5_ for Hispanic Californians. Two methods of comparing contributions to disparity in PM_2.5_ exposures from on-road vehicle fleet types shown for the most exposed racial-ethnic group, Hispanic Californians. First, we compare the absolute magnitude in contribution from each vehicle group (A and B); then, we compare the relative disparity in exposure to each vehicle group (C). (**A**) Absolute disparities in PM_2.5_ exposure from vehicles for Hispanic Californians relative to the overall statewide population declined between 2000 and 2019, consistent with the overall reduction in emissions (fig. S1) and population-weighted mean PM_2.5_ concentrations (Fig. 1). (**B**) Fractional contributions to the overall disparity that are attributable to each fleet type are estimated by normalizing the absolute contribution to disparity attributable to a single fleet type to the total disparity attributable to all on-road mobile sources. In each year, light-duty vehicle (LDVs) emissions are the dominant contributor to the disparately high exposures experienced by Hispanic Californians. (**C**) Disparities attributable to emissions of individual vehicle fleet types relative to the statewide average PM_2.5_ exposure attributable to emissions of that individual vehicle fleet. Note that heavy-duty vehicles (HDVs) especially disparately impact Hispanic Californians, even though HDVs are not the dominant contributor to overall emissions (fig. S1), PM2.5 concentrations (Fig. 1), or absolute disparities (C).

At the statewide average, we find that LDVs are the vehicle fleets with the largest aggregate impact on overall PWM PM_2.5_ exposures and absolute disparities. For example, considering Hispanic Californians, LDVs account for 65 to 70% of the 0.2 to 0.4 μg/m^3^ absolute disparity in PM_2.5_ exposure from on-road mobile sources (Fig. 3, A and B). Contributions to the absolute disparity from HDVs (16 to 24%), MDVs (9 to 14%), and all other vehicles (<5%) are substantially smaller. Considering the PWM distribution of PM_2.5_ by vehicle fleet type and race-ethnicity, we find broadly similar racial-ethnic distributions of exposure attributable to LDVs, HDVs, and MDVs, with Hispanic Californians receiving the highest exposures (fig. S5). Between 2000 and 2019, the fractional contributions to absolute disparity from individual vehicle fleet types were stable, likely reflecting the roughly constant distribution of vehicle activity patterns by vehicle fleet.

From here on, we focus our discussion on LDVs and HDVs, which, in combination, account for >80% of exposures and absolute disparities attributable to on-road sources (see fig. S7 for detailed results for other fleets). The dominant influence of LDVs on exposure holds across racial-ethnic groups and for residents of overburdened communities (figs. S9 and S10), but with different overall magnitudes of exposure for different subpopulations. This result likely arises for two reasons. First, LDVs dominate the overall emissions of PM_2.5_ and its precursors. On the basis of the CARB emissions inventories used, LDVs contribute most of the NH_3_ and VOC emissions (70 to 95% of NH_3_ and >80% of VOC) from vehicles, which account for ~44 to 56% of total PM_2.5_ exposure from vehicles. LDVs and HDVs contribute more similarly to primary PM_2.5_ (23 to 45% LDV and 29 to 56% HDV) and NO*_x_* (35 to 48% LDV and 36 to 43% HDV) emissions, and these species contribute the remaining ~44 to 56% of total PM_2.5_ (figs. S11 to S14). Primary PM_2.5_ emissions are more weighted toward non-exhaust emissions in recent years, especially for LDVs (figs. S11 to S14). Second, LDV emissions are more concentrated near population centers than other vehicle fleets, so LDVs result in a substantially higher-than-average exposure impact [fig. S15, population-weighed exposure per ton of annual emissions (metric: micrograms per cubic meter); this metric is directly related to intake fraction, e.g., (*61*–*63*)].

While the high activity of LDVs causes a higher aggregate impact on disparity, HDVs stand out as the fleet type whose emissions cause the most disparate impact on Californians of color. As a complement to apportioning the overall absolute exposure disparity to emissions from individual vehicle types (i.e., largest aggregate impact), in Fig. 3C, we also consider which vehicle fleet types have an especially disparate impact on specific racial-ethnic groups (largest relative impact regardless of the magnitude of emissions) relative to the statewide population. For example, the relative disparity caused by HDVs for Hispanic Californians (range: 16 to 17%) was larger than the relative disparity caused by LDVs (range: 11 to 14%). This difference in impacts by fleet type is consistent with recent traffic equity modeling, which demonstrated that the majority of Californians of all race-ethnicity are exposed to high annual average daily traffic from LDVs, but Californians of color are disproportionately exposed to higher annual average daily traffic from HDVs (*64*). Another useful metric for discussing the especially disparate impact of HDVs is the exposure inequality, defined by Demetillo *et al*. (*65*) as the percent difference in exposure between the most and least exposed racial-ethnic groups. On the basis of our results, the PM_2.5_ inequality for Hispanic Californians, relative to white Californians, increased from 37% in 2000 to 41% in 2019. This finding complements recent work that shows the importance of HDV emissions mitigation for reducing racial-ethnic disparity (*23*, *65*).

### Substantial heterogeneity in fleet-wise contributions at the community scale

**Fig. 4.**
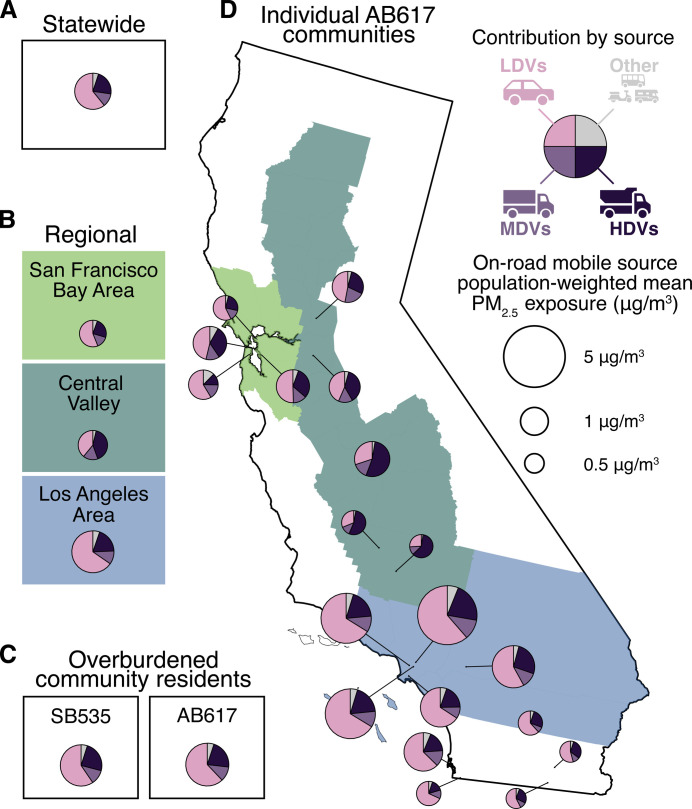
Spatial heterogeneity in contributions by fleet to mobile source PM_2.5_ exposure. Contribution to PM_2.5_ exposures from distinct vehicle fleets is shown at four spatial scales: (**A**) statewide, (**B**) three major regions, (**C**) residents of overburdened communities, and (**D**) for 19 individual communities designated by the state of California through the Community Air Protection Program (AB617; see fig. S2 for identification of each community). At each spatial scale, pie chart icons indicate the fractional contribution to exposure attributable to each vehicle fleet type, with icons scaled in proportion to the population-weighted mean PM_2.5_ concentration from all vehicle types. Light-duty vehicles contribute especially to mobile source PM_2.5_ exposures in Southern California, while the relative contribution from MDVs and especially HDVs is comparatively higher in the Central Valley and San Francisco Bay Area. There is considerable heterogeneity among AB617 communities in fleet contributions.

We find that there is substantial spatial heterogeneity in how different vehicle fleet types contribute to PM_2.5_ exposures. We compare modeled contributions by vehicle type at four spatial scales: statewide (Fig. 4A), regional (Fig. 4B), within overburdened communities (Fig. 4C), and community scale (Fig. 4D). Whereas the previous section and Fig. 4C evaluate aggregate exposure and disparity across all AB617 overburdened communities, in Fig. 4D, we compare contributions to exposure and disparity within individual overburdened communities. The primary goal of this analysis is to highlight the heterogeneity among diverse communities in how vehicle fleets contribute to PM_2.5_; our estimates are not meant to precisely capture community-scale pollution concentrations. As with any emissions inventory, modeled concentrations are much more precise with locally validated, site-specific information that has been observationally verified (*66*). To complement our high-level approach to understanding the heterogeneity in source contributions, future community-specific analyses could use higher spatial resolution modeling tools and local emissions data to better represent the lived experience of individual communities.

On average, the Los Angeles area and its AB617 overburdened communities have high PWM exposures and high contributions from LDVs (>60%). In the Central Valley, while the PWM exposures are lower, the contributions from HDVs are substantially higher (e.g., ~60% in Arvin/Lamont). The diversity in fleet contributions to individual communities showcases the importance of community-specific emissions reduction planning. While a community with a high share of LDVs, for example, might benefit more from policy actions that directly reduce those emissions (e.g., more electric bus routes and street conversion to bicycle paths), different strategies may be more appropriate for a community dominated by HDVs (e.g., additional diesel fuel emissions limits, truck electrification, and low emissions zones). These differences likely arise from differences in spatial distributions of sources relative to residences and the magnitude and mixtures of vehicle activity that occur at the community scale. In sum, our results support the approach of enabling communities to identify and mitigate the largest contributors to local exposures and disparities.

### Validation, limitations, and implications for future research

Multiple lines of evidence suggest that our core qualitative results align with available observational evidence. A relative strength of our modeling approach is that it allows us to model temporal changes at a sufficiently high spatial resolution that we can estimate exposure disparities attributable to individual source categories. In contrast, a detailed longitudinal record of in situ observations is not available at sufficient spatial resolution to permit a rigorous assessment of how disparities in exposure to traffic-related PM_2.5_ have evolved. Nonetheless, CARB’s analyses of ambient monitoring data from 1990 to 2014 align qualitatively with our results. These analyses of monitoring data indicate declining concentrations of diesel PM, PM_2.5_, and NO_2_ but with persistent relative and absolute disparities for the relatively sparse network of sites located in overburdened communities (*60*).

As additional points of comparison for our modeled results, we examined datasets of finely resolved satellite observations and empirical model predictions. These datasets afford the ability to consider changes in exposure and disparity for the entire state (see Supplementary Text) (*58*, *59*). In fig. S3, we compare our analyses with changes in total PM_2.5_ (only moderately influenced by vehicles) and NO_2_ (strongly influenced by vehicles). Considering PWM concentration changes from 2000 to 2019, our estimated PWM PM_2.5_ from on-road vehicles declined at a broadly similar rate (~65%) compared to the results from a high-resolution empirical model of PWM NO_2_ spatial patterns (~55%). Our estimates of the racial-ethnic ordering of vehicle-emitted PM_2.5_ exposures and disparities closely match those from the total PM_2.5_ and NO_2_ datasets. Crucially, our finding of temporally persistent relative disparities in exposure to PM_2.5_ from on-road sources (Fig. 1B) is consistent with highly stable patterns of relative disparity in total PM_2.5_ and NO_2_ for Californians of color (fig. S3). Furthermore, we find that the magnitude of our estimate of traffic-related PWM PM_2.5_ is consistent with the on-road vehicle contribution from previous modeling and in situ source apportionment studies in California (*67*–*72*). In combination, these supporting lines of evidence reinforce our key qualitative conclusion that exposures from mobile sources have decreased while relative disparities in exposure have persisted.

It is worthwhile to consider possible uncertainties, biases, and limitations associated with our approach. Our modeling framework is built around the InMAP reduced complexity model (*73*) and its associated InMAP source-receptor matrix [ISRM; (*28*)]. The computational efficiency of this model enabled us to interactively execute thousands of unique model runs representing distinct vehicle fleets for 20 individual years while maintaining a sufficiently fine scale (down to 1 km^2^) to capture spatially sharp exposure disparities (*1*, *3*). However, our modeling approaches have notable limitations. First, as with any atmospheric modeling, our results rest on the validity of underlying emissions inventories, including how they represent patterns over time and space [see, e.g., (*74*)], as discussed briefly below. Second, InMAP makes simplifying assumptions that can lead to somewhat higher bias than traditional chemical transport models (CTMs), which model the underlying atmospheric chemistry and dynamics with higher fidelity. One such simplification in our model is a linear approximation of nonlinear secondary aerosol chemistry. Third, the temporal resolution of our results is limited to annual average conditions; we do not quantify exposure disparities that occur on seasonal, diurnal, or shorter-than-annual timescales, which are also relevant (*75*)*.* In addition, our model does not capture sub-grid scale exposure gradients near roads; those gradients can occur and are important for exposure disparities at scales finer than 1 km (*76*). InMAP results are generally considered more robust for spatial aggregations of many grid cells (e.g., air basins and groups of overburdened communities), and less so for individual pixels or neighborhoods (*73*). Last, our core analyses assign exposures based on a fixed residential address [which can misclassify exposures; see, e.g., (*77*)] and were estimated using a temporally static year 2010 US census dataset (selected as the midpoint year of our study).

Considering that our key results emphasize the persistence over time of disparities (especially relative disparities)—rather than absolute concentrations at specific locations—our overarching qualitative insights are likely to be robust. Relative disparities are principally determined by the interaction of fine-scale spatial patterns of demographics, roadways, and fleet activity and are less sensitive to the magnitude of emissions or concentrations. In the Supplementary Materials (the “Model uncertainty and sensitivity” section of Supplementary Text and figs. S16 and S17), we explore how possible biases in emissions estimates and model performance could affect our results. We first review the literature to constrain our understanding of the uncertainty from the state regulatory model of on-road mobile source emissions estimates (*50*, *74*). From previous work validating EMFAC’s on-road mobile source emission factors and a similar model’s activity-based spatial surrogates, we believe that our qualitative insight is unlikely to be affected by bias in the emissions inventory. We then perform four tests to evaluate the sensitivity of our analyses to potential biases in the emissions and model (fig. S16). A key insight is that within the range of expected model biases for InMAP, we find that the magnitude and ordering of relative disparities are only minimally sensitive. This result arises in part because relative disparities involve ratios of modeled concentration estimates. In addition, because we find meaningful disparities for each of the five modeled PM_2.5_ constituents, pollutant-specific model biases (representing, for example, a possible mischaracterization of the nonlinear chemistry in InMAP) are unlikely to strongly skew our results (see fig. S16). Likewise, this analysis implies that inventory biases that affect aggregate levels of emissions are unlikely to affect our core insights. We show that our results are robust against spatial biases in the emissions inventory by repeating our analysis with emissions from two independently derived, peer-reviewed emissions inventories (*78*, *79*) and coarser representations of our emissions inventory. Spatial emissions biases in the inventory could conceivably affect conclusions about relative disparities if they were much larger than what we explored in fig. S16. However, we consider this implausible given how closely our results align with disparity insights from high-resolution NO_2_ predictions (fig. S3). Nonetheless, because neither EMFAC nor InMAP is meant to authoritatively describe emissions and concentrations in individual model pixels, we ascribe our greatest confidence to overall patterns in space and time, but caution against overinterpreting results for specific communities or other small regions. Last, in a sensitivity analysis (fig. S17), we repeated our analysis with 2000 decennial census data. These analyses indicate that our core qualitative finding of highly persistent relative disparity was generally not sensitive to the selection of the census dataset. In particular, the choice of which demographic dataset is used minimally affected the PWM concentrations and relative disparities experienced by overburdened communities and individual racial-ethnic groups in California. However, future demographic shifts in the California population that substantially alter patterns of social segregation could meaningfully affect aggregate air pollution disparities at the state level. For example, if suburbs become more racially integrated and California’s population becomes increasingly diverse, then relative disparities could decrease as a function of demographic changes and not necessarily emissions mitigation.

Future research beyond the scope of this assessment could further corroborate our findings and build on our results. First, CTM simulations could usefully validate our core results, especially as they concern the behavior of secondary PM_2.5_ from vehicle emissions. Second, it would be helpful to quantify the effect of decades of vehicle emissions controls on other air pollutants that are relevant to the health of overburdened communities, including nitrogen oxides, diesel PM, and air toxics. Third, although in situ observations of TRAPs have historically not been available at a sufficiently high spatial resolution to systematically characterize changes in disparity, careful analysis of data at particular locations may be able to complement our statewide insights. Moreover, as hyperlocal measurements of TRAPs become more widespread, these types of observational studies may be more feasible in the future.

Last, because disparities in terms of health outcomes are also relevant to EJ and distinct from exposure disparities, analyses that quantify the complex interplay of emissions, exposures, and social, demographic, and epidemiological factors could explore the impact of vehicle emissions on environmental health disparities over time (*22*, *23*, *25*–*27*, *80*–*83*). Disparities in health outcomes are strongly influenced by social determinants of health (e.g., age, obesity, access to health care, and criminal justice) that have persisted over time and are independent of air pollution (*80*, *84*). Several recent studies show that Black and Hispanic Americans in the United States have a higher susceptibility to air pollution than non-Hispanic white Americans (*81*–*83*, *85*). Thus, a focus on disparities in exposure may underestimate or mischaracterize the ultimate disparities in health outcomes (*22*–*27*). While this study focused exclusively on exposure disparities, effective policies should address disparities in both exposure and health outcomes.

### Policy insights from California’s historical mobile vehicle control policies

We have demonstrated that while modeled PWM PM_2.5_ exposures and absolute exposure disparities attributable to on-road mobile sources have decreased over the past two decades across all population groups, relative disparities have remained at both the average and at the extreme ends of the exposure distribution for Californians of color and residents of overburdened communities. Emissions from LDVs and HDVs affect disparities in different ways. LDVs contribute the most to PM_2.5_ concentrations and absolute disparity, while emissions from HDVs most disproportionately expose people of color relative to other fleet types, thereby highlighting the importance of mitigating emissions from both vehicle types. Of the groups considered here, residents of AB617 communities in aggregate experience the highest levels of PM_2.5_ exposure from on-road vehicles, although PWM exposures for these residents have declined by more than 60% since 2000. There is substantial heterogeneity among AB617 communities in terms of the total exposure concentration and the relative contribution from each vehicle type.

Our finding of highly persistent relative disparities for Californians of color is disappointing but consistent with a growing body of literature on sectoral emissions policy. When policies reduce the overall emissions rate without substantially altering the pattern of where emissions occur, relative disparities in exposure can persist (*9*, *14*, *21*). In this vein, the findings from our retrospective analysis resonate with the results of studies that have prospectively modeled the potential future equity impacts associated with specific vehicle policies (e.g., heavy-duty truck electrification and zero-emission vehicle adoption). Consistently with those studies, we have found large absolute concentration changes in regions with the highest share of people of color, yet we nonetheless find a minimal reduction in the relative disparity for PM_2.5_ exposure (*14*, *23*, *25*, *52*, *86*). These results arise because the places with the largest concentration changes over time tend to be the places most affected by vehicles (fig. S8).

While the sustained inequity in PM_2.5_ exposure resulting from on-road mobile sources is problematic, California’s mobile source strategy has led to large aggregate reductions in emissions, exposure concentrations, and absolute disparities. Although the relative disparity in exposure to PM_2.5_ from on-road mobile sources is effectively unchanged for Californians of color and residents of overburdened communities, the PWM PM_2.5_ exposures caused by these mobile sources were reduced by approximately 64% for all demographic groups considered during our study. On-road mobile source controls have also reduced emissions from a broad suite of TRAPs (*87*) that are also of health concern. For example, statewide on-road emissions of carbon monoxide, nitrogen oxides, and diesel PM also decreased by ~75% (*38*, *39*). Ambient concentrations of these pollutants have declined substantially in absolute terms, especially at sites in overburdened communities (*60*). These results speak to the value of both aggressive mobile source control and a multi-pollutant mitigation strategy that considers multiple TRAPs at once. Future mitigation efforts should continue this approach to avoid the risk of unintended consequences of single-pollutant control strategies (*52*). Despite this success, it is likely that relative disparities for other pollutants with similar spatial patterns of on-road emissions have persisted. For example, NO*_x_*, for which on-road sources contributed 57% of total statewide emissions in 2000, is considered. From 2000 to 2019, our assessment of high-resolution empirical model predictions shows a ~55% decrease in PWM NO_2_ but large and moderately increasing relative disparities by race-ethnicity (fig. S3).

It is useful to consider the implications of our retrospective assessment for California’s current policy efforts that focus heavily on eliminating exhaust emissions across the on-road fleet through a combination of electrification and—in the case of HDVs—hydrogen. For every year of the study, approximately 90% or more of the PWM PM_2.5_ exposure (and absolute exposure disparity) is attributable to exhaust emissions and approximately 80% or more is attributable to secondary formation from precursor exhaust emissions (figs. S11 to S14). Because California’s policies contemplate eliminating exhaust emissions, this result implies that future vehicle electrification has the potential to substantially reduce exposures and absolute disparity. Nonetheless, PWM exposure to non-exhaust primary PM_2.5_ emissions (i.e., brake and tire wear) increased somewhat from 2000 to 2019 (fig. S18), while relative disparities from non-exhaust primary PM_2.5_ remained effectively constant. Non-exhaust emissions would not be fully eliminated through electrification and could conceivably be exacerbated by increases in vehicle mass (*88*). Thus, future low levels of exposure from non-exhaust emissions (e.g., brake and tire wear) might still disparately affect people of color and residents of overburdened communities.

Our results suggest that relative disparities in exposure will persist without a paradigm shift in transportation policy. Some policy approaches have the potential to reduce not only aggregate levels of exposure but also relative disparities. For example, creating low emissions zones or promoting a mode shift away from private automobiles (e.g., dense public transit networks and bike lane infrastructure) could be more likely to reduce exposure disparities from the on-road vehicle fleet than statewide fleet-specific emissions controls, while also improving air pollution throughout the system (*89*). Without systemic changes to transportation infrastructure, it seems possible that these relative disparities could persist even in a future, lower emission scenario. Conversely, by strategically accelerating emissions reductions, such as vehicle electrification efforts, with deployment emphasizing overburdened areas, EJ communities could achieve substantial short-term reductions in relative exposure disparity.

While we have focused on one sector within California, our findings contribute to an emerging body of EJ research indicating that to reduce relative disparities in exposure, the policy must not only continue a trend of emissions reduction but also target the disparate geographical distribution of emissions in overburdened communities. While we focused on California as a case study, it is possible that these general findings would apply across the United States, as most state and national approaches broadly have mirrored California’s, with a strong focus on emission rate reductions. Our work provides a compelling illustration of how a highly successful emissions reduction strategy does not necessarily reduce relative disparity in exposures (*20*, *21*). More research is needed to identify the specific suite of strategies that can deliver a “triple win” for climate, health, and equity goals. We hypothesize that particularly effective strategies may go beyond aggregate emission rate reductions by ameliorating the inequitable spatial distribution of where activities and emissions take place. Thus, future work could explore the environmental equity impacts of potential policy actions and public investments that fundamentally change transportation infrastructure.

## MATERIALS AND METHODS

### Emissions estimates

We obtained estimates of mobile emissions in California from CARB’s EMFAC model (version EMFAC 2021 with MPOv11) for calendar years 2000 through 2019 (*38*). The EMFAC model uses detailed California-specific data to estimate emissions by year and fleet and has been approved by the US EPA (*53*). Estimated emissions were spatially allocated to a 1 km–by–1 km grid using surrogates developed by CARB and CARB’s Spatial and Temporal Allocator (ESTA) model. The ESTA model uses spatial surrogates that are derived from link-level traffic measurement data combined with population estimates and spatial information about idling locations, rest stops, and distribution centers (*90*). The resulting dataset contained spatially resolved annual total exhaust, evaporative, brake wear, and tire wear emissions for primary PM_2.5_ and four precursor species: NO*_x_*, VOC, NH_3_, and SO_x_. EMFAC2021 reports results for 54 vehicle categories and five fuel types (gasoline, diesel, natural gas, plug-in hybrid, and electric). Emissions for this analysis were binned into three main vehicle groups: LDVs, MDVs, and HDVs, with all other vehicle types (including motorcycles, motorhomes, and buses) grouped together as “Other” (table S1). Fleet information is derived from detailed data from the California Department of Motor Vehicles, the California Highway Patrol, the International Registration Plan Clearinghouse, and the National Transit Database (*38*). EMFAC is therefore capable of providing a reasonable representation of distinct activity and emissions patterns for specific vehicle fleets.

### Estimates of air concentrations

We modeled annual average PM_2.5_ concentrations attributable to vehicle emissions in California using the ISRM (*15*, *28*, *73*). The ISRM was developed from the US InMAP, which used WRF-Chem simulations and US Environmental Protection Agency National Emissions Inventory emissions estimates for 2014. The national version of InMAP was sampled on a population-weighted, variably sized grid (*n* = 21,705; 1 km to 48 km) for the state of California (*15*). Approximately 74% of grid cells are the finest resolution, with a population-weighted average grid size of 2.4 km (urban: 1.2 km; rural: 7.4 km). The gridding algorithm ensures that no cell larger than 1 km contains more than 20,000 people or a census block group with a population density higher than 2500 people/km.

The ISRM relates, for the *n* = 21,705 grid cells in California, marginal changes in ground-level concentration in every grid cell to marginal changes in emissions in every cell. Because this work only evaluates impacts from on-road mobile sources, all concentrations were estimated using the ground-level (i.e., 0 to 57 m above ground) layer.

### Open-source tool: ECHO-AIR

Air pollution modeling, even with reduced complexity modeling tools such as InMAP, can have major accessibility barriers for non-specialists. For the present analysis, we developed an open-source Python-based pipeline that streamlines exposure concentration and health impact analyses. The resulting system, called Estimating Concentrations and Health Outcomes – Automated ISRM Resource (ECHO-AIR), aims to lower barriers to entry for rapid estimation of PM_2.5_ exposure and health assessments.

Executing ECHO-AIR for analyses in California requires only estimates of emissions, which can be input as ArcGIS-compatible shapefiles or comma-separated value files. ECHO-AIR is modular, enabling users to use any ISRM, population data, and health input data, so long as they are formatted correctly ECHO-AIR is managed through a public GitHub repository to ensure transparency, maximize usability, and perform routine model upgrades and maintenance (see Supplementary Text for details).

### Population estimates

We obtained population data for the years 2000 and 2010 from the decennial US census for California from the National Historic Geographic Information System (NHGIS) database version 16.0 (*91*). Population estimates were queried at the tract level by age, race, and Hispanic origin. Consistent with prior literature (*4*, *8*, *9*), racial-ethnic categories were estimated as follows: The population count for Hispanic Californians was defined as Californians of any race who were of Hispanic origin; Californians who are not of Hispanic origin and are Black or African American alone, Asian alone, or white alone were defined as Black, Asian, and white Californians, respectively; all other Californians were included in the other category.

### Exposure assessment and disparity analysis

We estimated statewide group-level exposures to annual average PM_2.5_ as PWM concentrations, consistent with the air pollution disparity literature (*3*, *8*, *9*, *19*). For the metrics below, we consider only on-road mobile source exposure (i.e., we neglect contributions from other source types unless explicitly stated otherwise). To estimate exposure to PM_2.5_ for each year, we calculate geographic intersections between the 2010 census tract boundaries and the gridded concentration estimates. Population is down-sampled based on area apportionment; concentration estimates are assumed to be constant throughout the grid cell. Exposure concentrations are calculated at the smallest geography possible (e.g., polygon intersection of census tract and ISRM grid cell).

The PWM exposure is estimated by multiplying the annual average PM_2.5_ concentration by the population of the demographic group of interest within that grid cell, summing across all grid cells, and dividing by the total population

$${\text{PWM}}_{k}=\frac{\sum_{i=1}^{n}P_{i,k}\times C_{i}}{\sum_{i=1}^{n}P_{i,k}}$$

where PWM*_k_* is the PWM exposure concentration for group *k* across *n* grid cells, *P_i,k_* is the population of group *k* in grid cell *i*, and *C_i_* is the concentration of PM_2.5_ in grid cell *i*. Equity was assessed using the absolute and relative disparities at the PWM. The absolute disparity (*D_A,k_*) is defined as a demographic group’s PWM exposure (*PWM_k_*) subtracted by the statewide PWM exposure (PWM*_T_*)

$$D_{A,k}={\text{PWM}}_{k}-{\text{PWM}}_{T}$$

Relative disparities (*D_R,k_*) are estimated as the absolute disparity divided by the statewide PWM exposure to mobile sources

$$D_{R,k}=\frac{\left({\text{PWM}}_{k}-{\text{PWM}}_{T}\right)}{{\text{PWM}}_{T}}=\frac{D_{A,k}}{{\text{PWM}}_{T}}$$

Because the ISRM is a linear model and the absolute disparity is an arithmetic equity metric, absolute disparities can be apportioned to individual source categories to find a relative contribution to the absolute disparity. Thus, the fractional contribution of a source’s emissions to a group’s exposure is estimated as follows

$$f_{j,k}=\frac{D_{A,j,k}}{D_{A,t,k}}$$

where *f_j,k_* is the fractional contribution from source *j* on the exposure and disparity for group *k*, *D_A,j,k_* is the absolute disparity from source *j* for group *k*, and *D_A,t,k_* is the absolute disparity for group *k* from all on-road mobile sources.

## Supplementary Materials

### This PDF file includes:

Supplementary Text Figs. S1 to S18 Tables S1 to S6 References
