# Supplementary material for: PM2.5 exposure disparities persist despite strict vehicle emissions controls in California
Source: Sci Adv. 2024 Sep 11;10(37):eadn8544. doi: 10.1126/sciadv.adn8544 (PMC11389777; doi:10.1126/sciadv.adn8544)
Supplement: Supplementary file 1 — Supplementary Text Figs. S1 to S18 Tables S1 to S6 References [file sciadv.adn8544_sm.pdf]

Science Advances
AAAS

Supplementary Materials for

$PM_{2.5}$ exposure disparities persist despite strict vehicle emissions controls in California

Libby H. Koolik *et al.*

Corresponding author: Joshua S. Apte, apte@berkeley.edu

Sci. Adv. **10**, eadn8544 (2024)
DOI: 10.1126/sciadv.adn8544

This PDF file includes:

Supplementary Text
Figs. S1 to S18
Tables S1 to S6
References

Supplementary Text

Text S1. ECHO-AIR Model Details

This work uses a novel open-access pipeline called **E**stimating **C**oncentrations and **H**ealth **O**utcomes **A**utomated **I**SRM **R**esource (ECHO-AIR) to estimate $PM_{2.5}$ exposure concentrations. We developed ECHO-AIR to reduce barriers of entry in air pollution modeling. Our goal in creating this pipeline was to develop a resource that required little-to-no computer programming to empower users from government, industry, academia, and environmental justice groups to estimate $PM_{2.5}$ concentration and health outcomes from emissions sources.

The ECHO-AIR pipeline works by a series of two modules. First, ECHO-AIR estimates annual average change in $PM_{2.5}$ concentrations as part of the Concentration Module. The Concentration Module utilizes the InMAP Source Receptor Matrix (ISRM), which links emissions sources to changes in receptor concentrations. A second and optional Health Module, not used here, permits estimation of excess mortality based on user-selected concentration-response functions.

ECHO-AIR is developed in Python and managed through Github, enabling the pipeline to be entirely open-access. The code can be downloaded by cloning the repository: https://github.com/echo-air-model/echo-air

Detailed documentation has been written to explain the methodology, to walk the user through setting up and running the model, and to describe the input and output files. The documentation can be viewed via web browser here: https://echo-air-model.github.io/.

Text S2. Validation and Comparison to Other Estimates

Here, we aim to validate our results with previously published observations and model data. The observational record lacks the spatial, temporal, and chemical information to track the long-term evolution of primary and secondary $PM_{2.5}$ contributions attributable to vehicles only across California at sufficient spatial resolution to quantify disparities. Accordingly, we interrogate the plausibility of our results by considering three fundamental questions that we can individually validate with independent peer-reviewed estimates of ambient $PM_{2.5}$, NO_2, and diesel particulate matter. First, are the changes we find in statewide PWM exposures from on-road mobile sources consistent with California's ambient total $PM_{2.5}$ and NO_2 concentrations? Second, are the changes in exposure and relative disparity in exposure by race-ethnicity comparable with estimated disparity by race-ethnicity for total $PM_{2.5}$ and NO_2? Finally, are the changes we model in overburdened communities consistent with state regulatory monitoring in these communities?

For this discussion, we analyze two external datasets and briefly review observations from the literature. The first dataset is the annual mean $PM_{2.5}$ concentration derived from satellite observations gridded at 0.01° by 0.01° (average ~1 km^2 in California, *58*).The second dataset is from the Center for Air, Climate, and Energy Solutions (*59*), which predicts concentrations of $PM_{2.5}$ and NO_2 via an empirical (land use regression) model with high fidelity as compared to monitoring sites (R^2 = 0.84 and 0.81 for $PM_{2.5}$ and NO_2, respectively). NO_2 is a useful analog for mobile source $PM_{2.5}$, as on-road mobile sources are a major source of NO_x emissions in California (in year 2000, 58% of NO_x came from on-road sources) (*39*). In contrast, on-road mobile sources are only a small fraction (<10%) of California's estimated primary $PM_{2.5}$

emissions (*39*), and a larger, but not dominant, source of total $PM_{2.5}$ *concentrations* according to source apportionment studies (*67–72*).

We first contextualize the magnitude of our on-road mobile source PWM $PM_{2.5}$ concentration (relative to total PWM $PM_{2.5}$) against evidence from the literature. Our estimates of on-road mobile source PWM $PM_{2.5}$ represent approximately ~10-20% of the total PWM $PM_{2.5}$ derived from satellites or empirically modeled (*58*, *59*). This result is broadly in line with the available evidence from other modeling studies and in-situ source apportionment studies, which generally attribute ~10-25% of ambient $PM_{2.5}$ to mobile sources depending on the setting and study year (*67–72*).

Next, we find broadly similar temporal changes indicating sharply reduced concentrations but persistent relative disparity when considering these two gridded datasets. We recreate the analysis of PWM concentration over time (*cf.* Fig. 1a) using satellite derived $PM_{2.5}$ (Fig. S3A) and the empirically modeled $PM_{2.5}$ (Fig. S3B) and NO_2 (Fig. S3C) estimates. The reduction in total statewide PWM $PM_{2.5}$ (~45% reduction based on satellite and empirically derived estimates) is less than the reduction in our estimated mobile-source reduction (~65% reduction). This result makes sense – models and in-situ source-apportionment observations alike indicate that on-road mobile sources contribute only a fraction of ambient $PM_{2.5}$ in California, but mobile-source emissions of $PM_{2.5}$ and its precursors have declined more rapidly. In contrast, the magnitude of empirically modeled PWM NO_2 (~55% reduction) more closely resembles our result for the mobile source $PM_{2.5}$ reduction; this finding is reasonable given that mobile sources account for a substantially larger fraction of ambient NO_2 than ambient $PM_{2.5}$.

We then computed relative disparities from modeled and remotely sensed measurements of total $PM_{2.5}$ and NO_2 (Fig. S3B) to provide a point of comparison with Fig. 1b. Qualitatively, these results align closely with our core finding: relative disparities by race/ethnicity for total $PM_{2.5}$ and NO_2 persisted over the 2000-2019 time period. Considering the two $PM_{2.5}$ datasets, we found slight reductions in relative disparity (e.g., reduction from ~9-10% to 7-8% for Hispanic Californians from 2000 – 2019). For empirically modeled total NO_2, relative disparities by race-ethnicity increased slightly (e.g., for Hispanic Californians, increasing from 14 to 15%), which is broadly consistent with our finding of modestly increasing disparities for $PM_{2.5}$ from mobile sources (from 12-14%). Overall, the similarity between the relative disparity in exposure to NO_2 from the empirical model and the relative disparity in exposure to on-road mobile source $PM_{2.5}$ we estimate here instills confidence in our finding.

As a final point of comparison for overburdened communities, we consider a 2016 California Air Resources Board report on air quality in overburdened communities (*60*). Regulatory measurements of elevated concentrations of diesel particulate matter, $PM_{2.5}$, and NO_2 in overburdened communities between 2000 and 2014 support our finding that there is sustained disparity in overburdened communities during our study period (*60*). Additionally, they estimated an approximate 65% reduction in diesel particulate matter concentrations in overburdened communities from 2000 to 2014. While we do not explicitly model diesel particulate matter, we can approximate exposure to diesel particulate matter by isolating our estimate of PWM exposure to exhaust-only primary $PM_{2.5}$ from HDVs. For members of overburdened communities, our results are generally consistent with the findings from observations. From 2000-2014, our modeled results show PWM $PM_{2.5}$ reductions of 73% and 72% for AB617 and SB535 residents, respectively. In summary, available data from CARB regulatory monitoring supports our conclusions for overburdened communities.

Text S3. Model Uncertainty and Sensitivity

While the previous section validates our results against previous work, here we aim to discuss the potential uncertainties and bias associated with our modeling approach. The results and conclusions in this work are based on modeled concentrations from the ISRM (*28*, *73*) using state regulatory emissions modeled from EMFAC (*38*). InMAP and the ISRM have been validated against measurement and comprehensive chemical-transport models to adequately estimate population-weighted mean exposures (*28*, *73*). However, as with any model, results are potentially influenced by uncertainty, error, and bias. In this section, we discuss the sensitivity of our finding of persistent disparity to error and bias in the emissions estimates, reduced-complexity model, and population dataset.

Emissions Inventory Uncertainty and Sensitivity

With respect to the emissions inventory, while EMFAC is the state regulatory model for on-road mobile source emissions, emissions estimates can introduce error and bias that could influence our results (*74*). For completeness in this discussion, we decompose the emissions uncertainty in magnitude (relevant for our finding that population-weighted mean exposure has decreased for all Californians) and in space (relevant for our finding that disparities have persisted). Toward validation of our estimated changes in emissions over time, Yu et al. (*50*) found that EMFAC reasonably predicts the decrease in mobile-source NO_x emissions in California for gasoline and diesel vehicles when compared with a fuel-based inventory. While the spatial distribution of emissions from EMFAC have not been formally validated in the literature, the spatial surrogates used to allocate emissions to the 1 km modeling grid use carefully compiled and highly resolved data from other California state agencies. To help constrain uncertainty in our results due to potential spatial inaccuracies in emissions allocation, we consider analogous mobile source inventories with activity-based spatial surrogates. A study of a similar on-road mobile source emissions model (Neighborhood Emission Mapping Operation, NEMO), for example, found high overall spatial correlation but potential underestimation of emissions inequality between satellite-derived estimates of NO_2 and modeled NO_x emissions (*74*). In Fig. 16A, we repeat our analysis using two independently derived and peer-reviewed emissions inventories: EDGAR (v6.1, *78*) and NEMO (v2017, *91*). As shown in Fig. 16A, while the relative disparities in exposure estimated using the coarser emissions from EDGAR are lower for all groups, the relative disparity in exposure is sustained. Similarly, the relative disparity in exposure when modeled with emissions from a single available year of data (2017) from NEMO are consistent with our overall findings. In Fig. 16B, we show that the high-resolution spatial emissions allocation are not overly influencing our results. We repeat our analysis after re-gridding the EMFAC emissions to make our inventory coarser in space. Even with 4 km resolution emissions estimates, we find that exposure disparities persisted. Additionally, while EMFAC could have biases in its spatial emissions allocation, we believe our overall assessment of relative disparities for $PM_{2.5}$ from on-road sources is robust, given the consistency in magnitude and direction of our results with disparities in empirically modeled NO_2 concentrations (Fig. S3). Therefore, while there may be bias or uncertainty in the emissions inventory, we do not expect it to qualitatively affect our conclusions.

Reduced-Complexity Model Uncertainty and Sensitivity

A useful framework for discussing the uncertainty of the reduced-complexity model decomposes the model accuracy into two important axes: fidelity of the overall magnitude of

exposure concentrations, and fidelity of the spatial distribution of the exposure concentrations. Because our results rely on the spatial relationships between population groups and exposure concentrations, inaccuracies in the magnitude of overall concentration will perturb the population-weighted mean concentrations but will not affect the exposure disparities. Accordingly, we focus on potential *spatial* inaccuracies in the model. To properly assess the effects of model error and bias on our conclusions, here, we perform two sensitivity analyses on our estimated relative disparities in exposure.

First, we show that our result is robust against potential spatial and demographically linked biases in the model (Fig. S16B). To do this, we iterate through each of the racial-ethnic groups and perturb the concentrations by ±18% (the mean fractional error for InMAP versus WRF-Chem, *73*) for cells where 50% or more of the population is of that specific racial-ethnic group. We then estimate the PWM for the individual group and the full population, from which we estimate the relative disparity. The relative disparity under the perturbed conditions for each group are shown as error bars in Fig. S16B. As shown in Fig. S16B, we find that even if model errors were spatially biased in a manner that was substantially correlated with the racial-ethnic population distribution, disparities persist. While there is now some overlap between the error bars for the relative disparity in exposure for Hispanic, Black, and Asian Californians, relative disparities between Californians of color and white Californians persist, even when concentrations are underestimated in communities of color and overestimated in white communities.

Second, we test the sensitivity of our conclusion to potential errors in model performance associated with individual precursor pollutants. In Fig. S16C, we perform a systematic leave-one-out analysis on relative disparity for each precursor pollutant. To do this, we estimate PWM exposures for each racial-ethnic group from the total $PM_{2.5}$ concentrations (Fig. 1B) and each combination of four precursors. The relative disparity in exposure to total $PM_{2.5}$ is represented by a point, the minimum and maximum relative disparity caused by leaving out a single precursor create the error bars, and the relative disparity from secondary $PM_{2.5}$ only is indicated by a star. Again, we find that our results are robust to model error associated with any one of the precursor pollutants.

Sensitivity to Population Dataset

In our core analyses, we rely on static population data for year 2010, the midpoint of our 2000 – 2019 analysis time frame. As a sensitivity analysis (Fig. S17), we also considered how our results would change using a year-2000 population estimate, which reflects the only other year for which a reliable demographic estimate is available for California from the decennial census. From 2000 to 2010, California's Hispanic population grew by 28% while the white population decreased by approximately 5%, resulting in an overall larger share of the population identifying as Hispanic (32.4% Hispanic in 2000 and 37.6% Hispanic in 2010). Additionally, population migration occurred for all racial-ethnic groups in California, moving away from coastal cities toward more suburban or rural inland areas and decreasing overall segregation (*92*). We show that our finding that PWM exposure has decreased while relative disparity has persisted is recreated when using a static 2000 population dataset instead of a static 2010 population dataset. While the changes across time and demographic group are consistent, one difference between our main result (Fig. 1) and the result of this sensitivity test (Fig. S17) is the overall magnitude of the PWM. The higher estimated PWM using a static 2000 population dataset is consistent with the two aforementioned population changes that occurred in California

from 2000 to 2010. First, the lower PWM using 2010 population data is consistent with the population migration shifts away from cities, major roadways, and highways. Second, the increased fraction of California's population that is Hispanic affects the statewide total PWM. As the Hispanic share of the population increases, the statewide and Hispanic PWMs may converge over time, which will ultimately affect the relative disparity metric (for this sensitivity test, we observed only a modest impact of this effect). Nonetheless, comparing the sensitivity of our results to the exposure for members of overburdened communities, we find <2% difference in PWM and relative disparity across all years (e.g., PWM in 2000 for members of AB617 communities of 4.5 $\mu g/m^3$ using 2000 Census data and 4.4 $\mu g/m^3$ using 2010 Census data). Together, the similarities in exposure for members of overburdened communities and the shape of the PWM and relative disparity results suggest that the changes in the spatial distribution of emissions is a more important driver of disparity than the changes in the spatial distribution of the population.

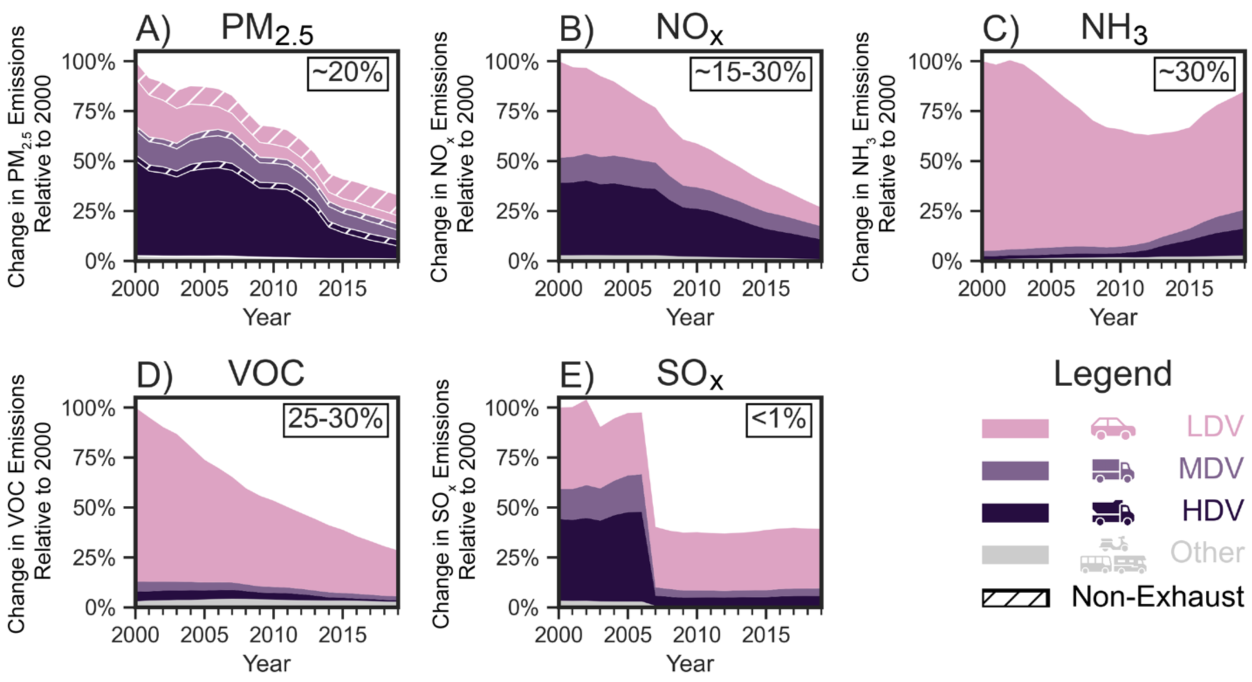

Fig S1. Emissions of $PM_{2.5}$ precursors by on-road mobile source fleet relative to 2000. Changes in total statewide emissions of each on-road mobile vehicle pollutant modeled as a precursor for total $PM_{2.5}$ concentration stratified by fleet type. Total statewide emissions are shown relative to the emissions in 2000. In the upper righthand corner of each panel, the approximate percent contribution to secondary $PM_{2.5}$ concentration is shown. For primary $PM_{2.5}$ emissions (A), emissions are split by mode using hatching to represent the non-exhaust fraction (brake wear and tire wear). Emissions of SO_x (E) are relatively unimportant, as the very low SO_x emissions results in negligible secondary $PM_{2.5}$. Emissions of primary $PM_{2.5}$, NO_x, and VOC have declined substantially from 2000 – 2019. Emissions of NH_3 (C) were declining in the first decade, but widespread catalytic conversion usage has led to a modest increase in emissions during the second decade of the study. For NH_3 and VOC (C-D), LDVs are responsible for the majority of statewide emissions; for $PM_{2.5}$ and NO_x, LDVs and HDVs contribute approximately evenly. The contribution of each precursor to total $PM_{2.5}$ concentration is explored further in Figs. S11-S14.

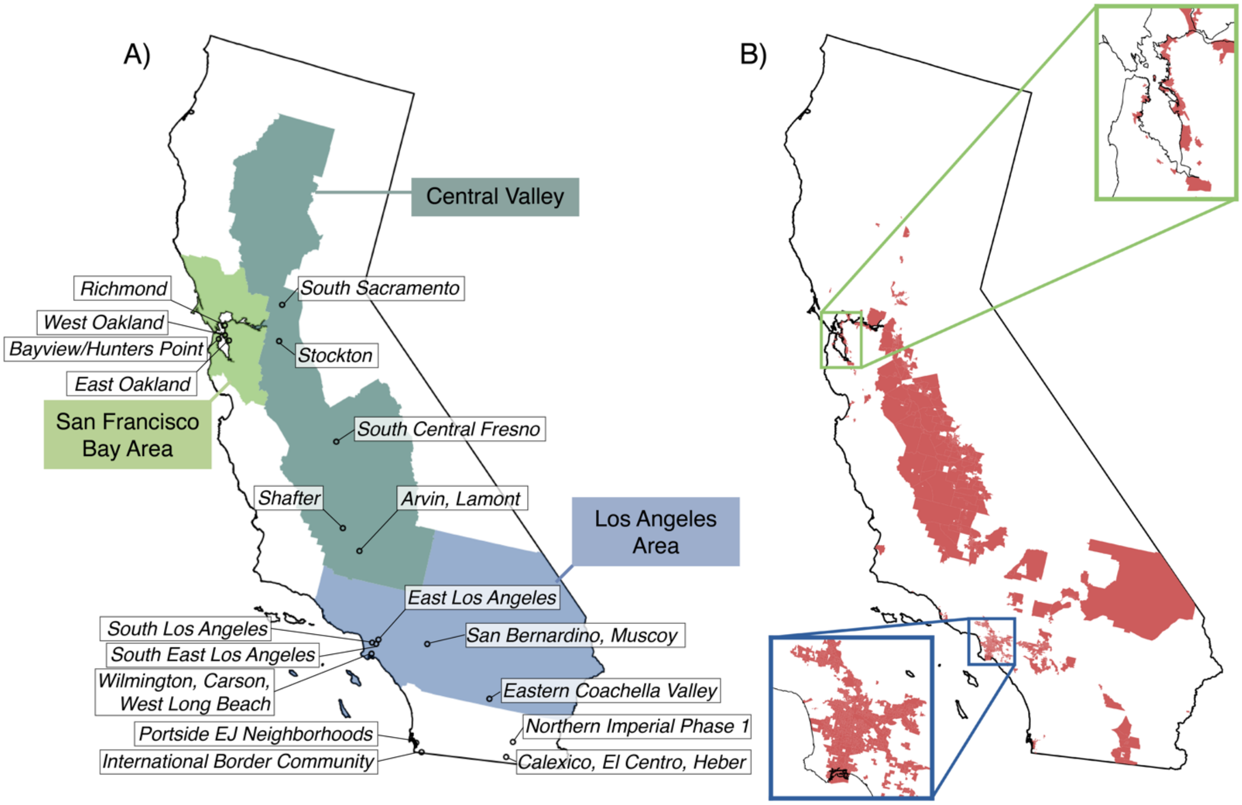

Fig. S2. Maps of overburdened communities in California.

The overburdened communities and geographic regions evaluated as part of this work are identified on two maps of California. (A) AB617 overburdened communities are marked by a circle with their names italicized. We also highlight three broad geographic regions (San Francisco Bay Area, Central Valley, Los Angeles Area), which we defined in terms of the counties associated with each region. (B) The SB535 overburdened communities are shaded in red. Inset maps show more detailed views into the San Francisco and Los Angeles metropolitan areas. While SB535 also includes tribal community areas, in this work we focus on the Census Tracts. All results computed for SB535 are at the statewide population-weighted mean. Because the population living in federally recognized tribal lands included in SB535 contribute a relatively small number of people, we do not anticipate that our results would change substantially upon inclusion of these areas.

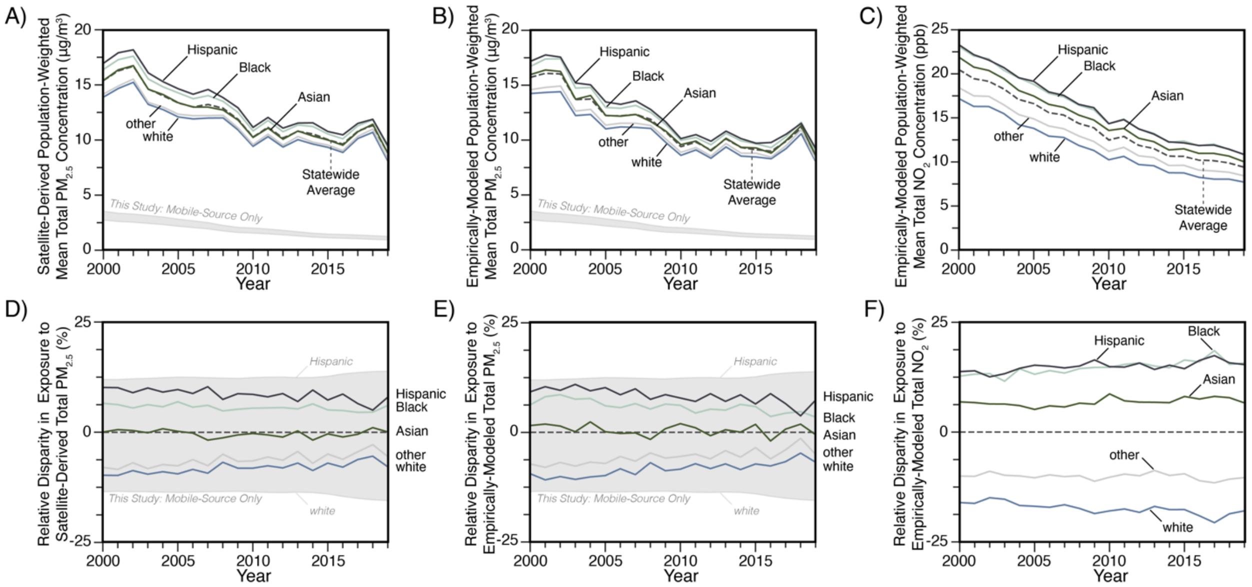

Fig. S3. Validation analyses to compare modeled results to other lines of evidence.
Here, Fig. 1 is reproduced with two additional data sets derived from satellite observations of $PM_{2.5}$ and an empirical model (land use regression) for $PM_{2.5}$.and NO_2. In panels (A) and (B), we estimate the PWM $PM_{2.5}$ exposure for each racial-ethnic group and the statewide population using estimates derived from satellite observations and empirical model, respectively. In panel (C), we estimate the PWM NO_2 exposure for each racial-ethnic group using estimates from the empirical model. Fig. 1B is reproduced for each of these datasets in panels (D) through (F). The gray shading in panels (A), (B), (C), and (D) superimpose the results by race-ethnicity from Fig. 1 directly. As discussed in the "Validation and Comparison to Other Estimates" supplementary text, the consistency in the trends in ambient PWM and relative disparity in exposure here support the robustness of our findings.

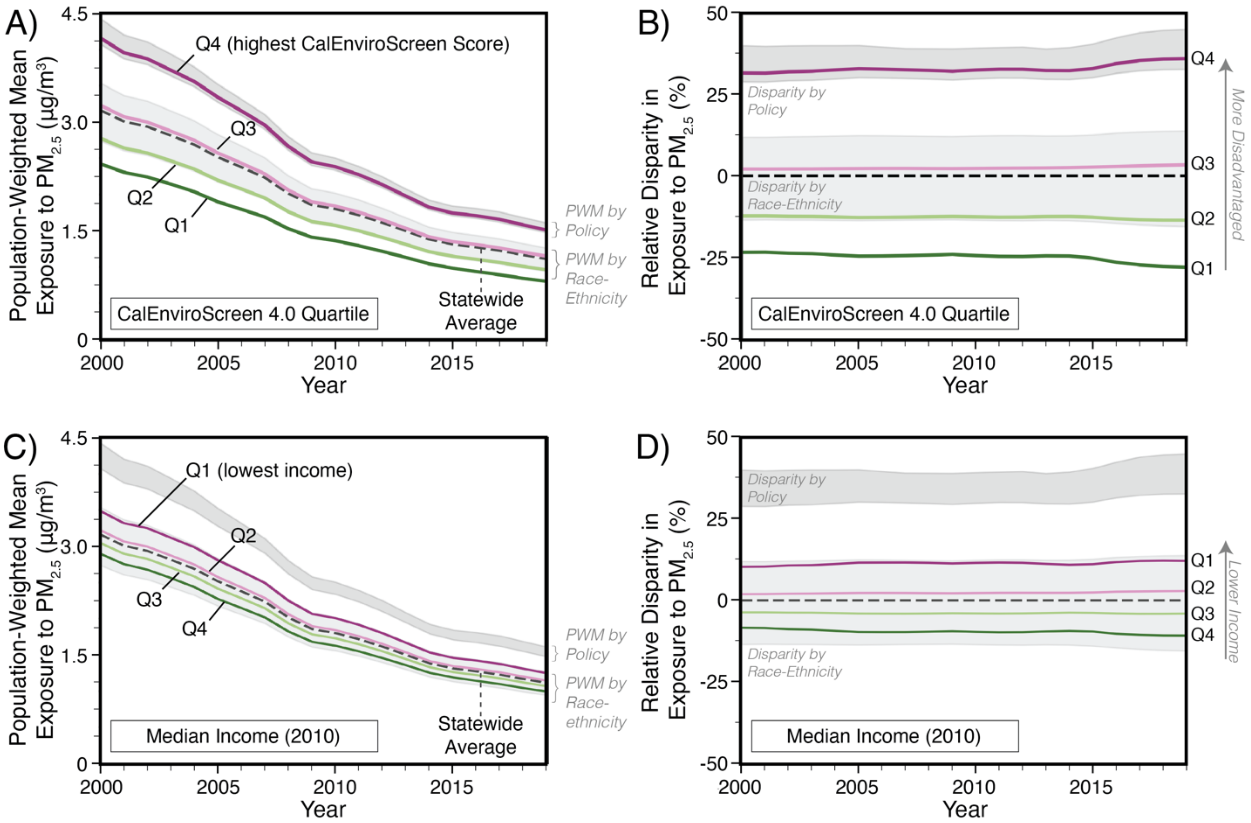

Fig. S4. On-road mobile-source $PM_{2.5}$ exposure and relative disparity in exposure for additional demographic variables.

Statewide population-weighted mean $PM_{2.5}$ exposure concentrations and relative disparity in exposure attributable to on-road mobile sources are shown for two additional demographic variables. In the top row, we show the (A) population-weighted mean $PM_{2.5}$ exposure concentration and (B) relative disparity for the four quartiles of CalEnviroScreen score relative to the two groupings of California's overburdened communities described in the main text (AB617 and SB535, "disparity by policy"). In the second row, we show these same metrics for four quartiles of California's income distribution (C, D). Income quartiles are defined as follows. Total population and median household income (adjusted to 2012 inflation) were queried at the block group level from the 2008-2012 American Community Survey. Income data were adjusted to 2010 using the Consumer Price Index. Block groups were then sorted by median income level and the percentile of the overall population was estimated as the cumulative number of people at that income level divided by the total population: < $40,892 Q1, $40,893-$58,452 Q2, $58,453-$82,842 Q3, and >$82,843 Q4. Each quartile represents approximately 9.3 million Californians. In each year, relative exposure disparities (B, D) for each group are computed in reference to statewide average $PM_{2.5}$ concentration attributable to on-road mobile sources.

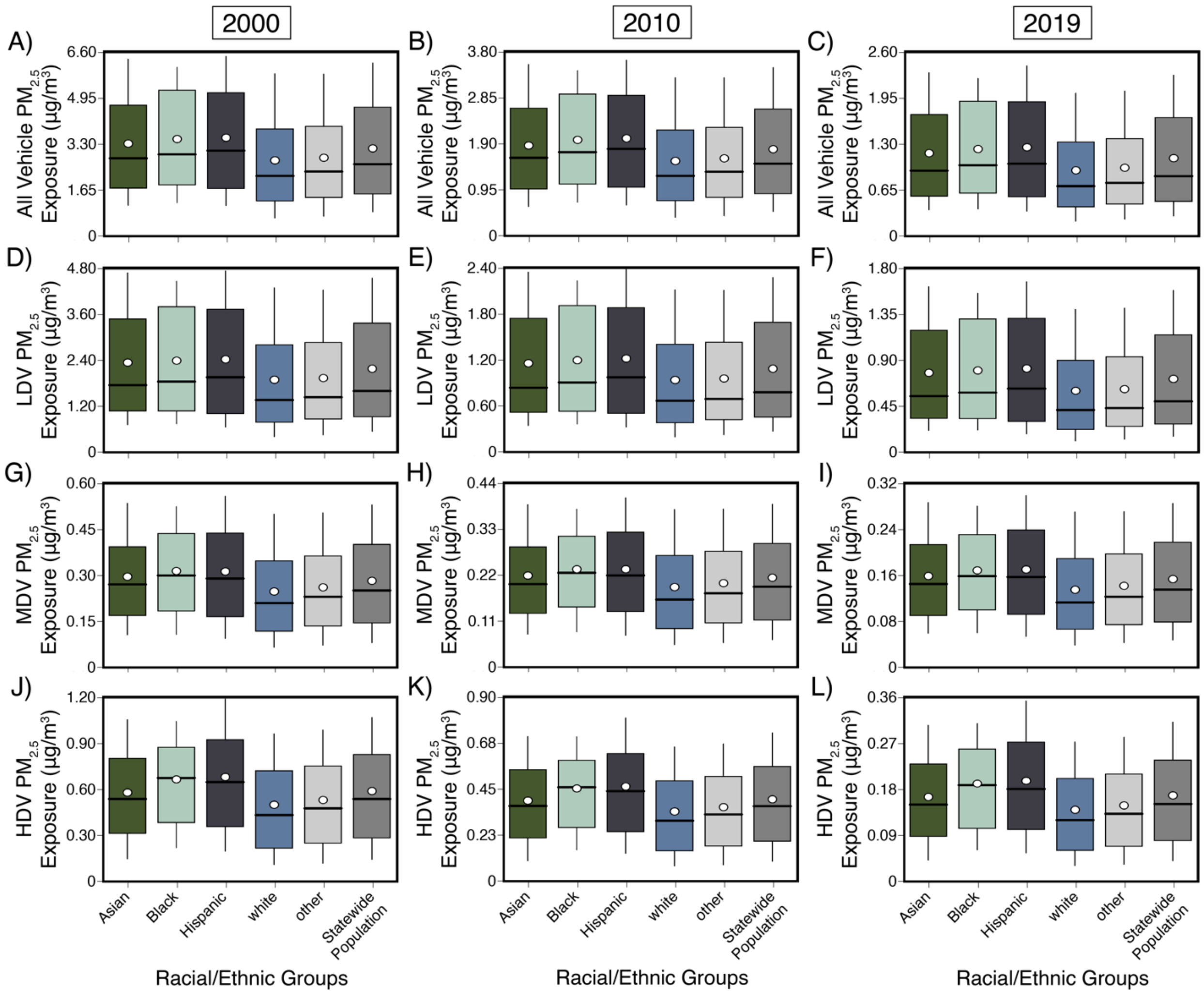

Fig. S5. Distributions of exposure to mobile-source $PM_{2.5}$ for racial-ethnic groups. Variation in $PM_{2.5}$ exposure distributions from on-road vehicle emissions for racial-ethnic groups in California in 2000 (lefthand column), 2010 (center column), and 2019 (righthand column). The rows represent each of the vehicle fleets: all vehicles (A-C), LDVs (D-F), MDVs (G-I), and HDVs (J-L). Boxplots are roughly centered around the means and indicate the following population-weighted statistics: median (central bar), mean (circle), 25th and 75th percentile (box), and 10th-90th percentiles (whiskers). Across the full exposure distributions and across time, white Californians experience among the lowest exposures to $PM_{2.5}$ from on-road mobile sources, while Hispanic and Black Californians experience among the highest exposures. Comparing the disparity at the most exposed (90th percentile) Hispanic Californians versus white Californians, the disparity is the most severe in exposure to HDVs. Overall, we note the roughly similar patterns by race/ethnicity for each vehicle fleet type.

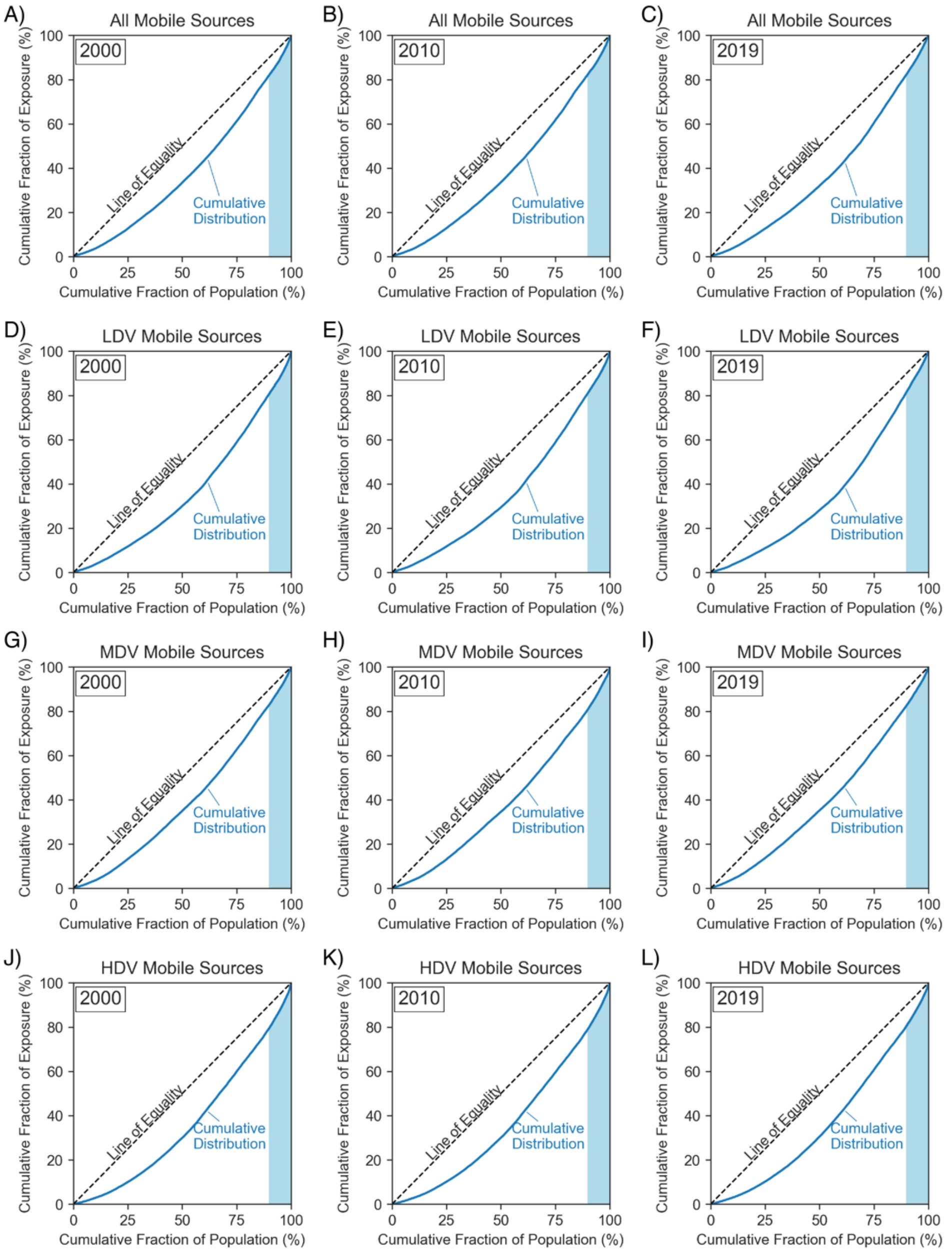

Fig. S6. Cumulative distributions of exposure.

Cumulative distributions of exposure are drawn for each vehicle fleet for each representative year. Cumulative distributions are estimated by finding the exposure concentration that corresponds to a uniform distribution of California's population, consistent with the methodology used to create Lorenz curves for income or wealth distributions. The leftmost column shows emissions for 2000, the center column represents emissions in 2010, and the rightmost column shows the result of emissions in 2019. Distributions are drawn for each fleet type: (A-C) all vehicles, (D-F) LDVs, (G-I) MDVs, (J-L) HDVs. Across all years and fleet types, the top decile of the population receives over 20 percent of the total population-weighted exposure and the top 25% of the population receives 35-40% of the total population-weighted exposure.

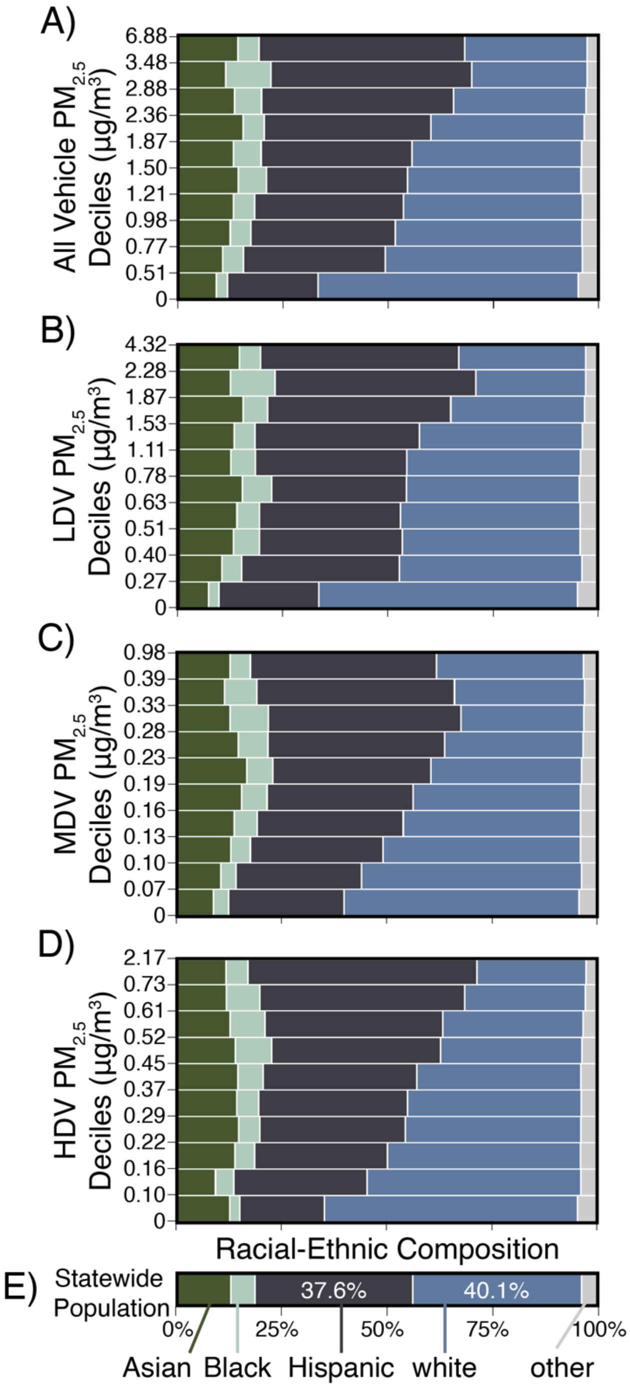

Fig. S7. Racial-ethnic population distribution by exposure decile across fleets in 2010.
The distribution of race-ethnicity for the population within each decile of (A) full fleet, (B) LDVs, (C) MDVs, and (D) HDV exposure. The statewide racial-ethnic composition is shown for comparison. As shown here, the results using emissions in 2010 do not substantially vary from the results using 2000 or 2019 emissions shown in Fig. 2, although the magnitude of exposure is different.

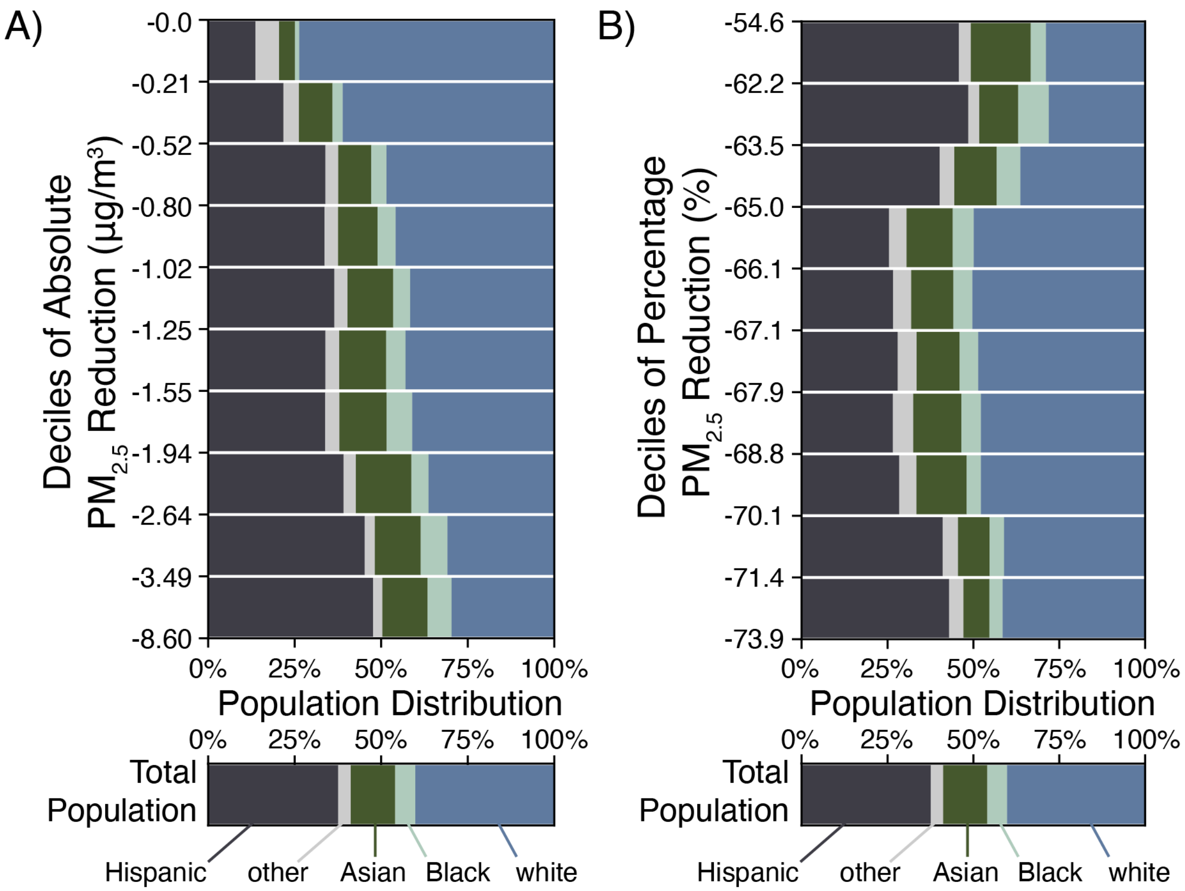

Fig. S8. Distribution of exposure reductions by race-ethnicity.

Statewide (A) absolute and (B) percent changes in $PM_{2.5}$ concentration from 2000 to 2019 from the full on-road mobile fleet are binned into deciles and the racial-ethnic composition of each decile of change is shown in comparison to the full statewide population. The left column (A) estimates absolute changes in concentration, following the methodology from recent vehicle electrification studies (e.g., *57*, *71*). (A) While the grid cells with the largest absolute reduction in concentration consist of more people of color than the statewide average (thus contributing to the reduction in absolute exposure disparity), (B) the smaller demographic differences in the percentage change in exposure highlight the persistent inequalities despite larger absolute reductions in communities of color. The difference between (A) and (B) arises in large part because the geographies with the largest absolute reductions in $PM_{2.5}$ exposure from on-road mobile sources started out with the highest initial levels of exposure in 2000.

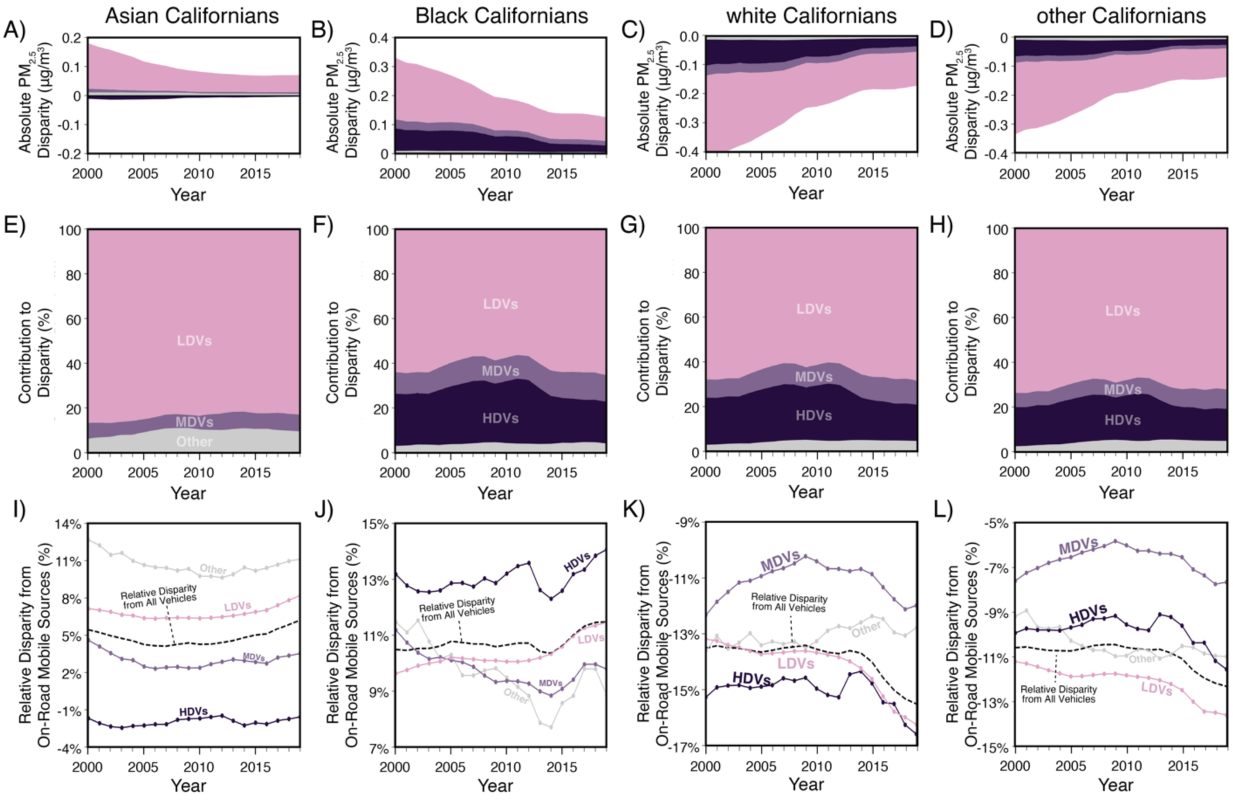

Fig. S9. Contributions to disparity in exposure to mobile-source $PM_{2.5}$ for each race-ethnicity.

This figure accompanies Fig. 3 (disparity for Hispanic Californians) by illustrating the disparity in exposure for each additional racial-ethnic demographic group. As with Fig. 3, we present three ways to disaggregate contributions from LDVs, MDVs, HDVs, and all other vehicles. The absolute disparity is estimated as the total population-wide on-road mobile-source exposure subtracted from the population-weighted mean exposure (A-D). The fractional contribution to disparity divides the absolute source contribution by the total absolute disparity (E-H). The relative disparity to each on-road mobile source compares an individual group's exposure to each individual vehicle type to the statewide average exposure to that vehicle group (I-L). Asian Californians (leftmost column) are the only racial-ethnic group that does not experience uniformly positive or negative disparity in exposure from all fleets. In other words, unlike all other racial/ethnic groups, Asian Californians are more exposed to LDVs, MDVs, and all other vehicles than the statewide average Californian but less exposed to HDVs. As a result, the contribution to disparity in panel (E) only shows the fractional contribution to the higher-than-statewide exposure disparity and HDVs are not included. For Black Californians (second column from the left), the relative contribution and source-specific relative disparity are very similar to those of Hispanic Californians (Fig. 3). White (second column from the right) and other (rightmost column) Californians are less exposed to all on-road mobile vehicles than statewide average, so note the opposite orientation of disparity in panels (C-D) and (K-L).

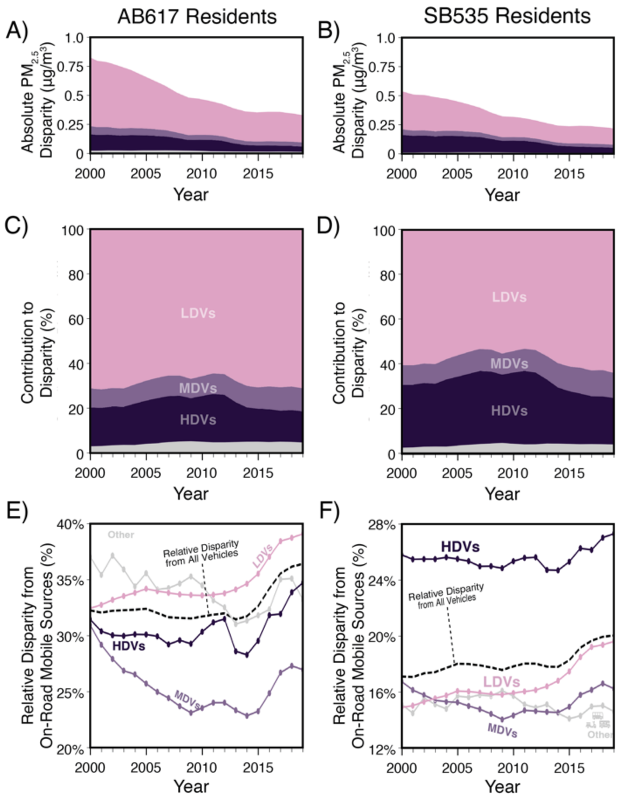

Fig. S10. Contributions to disparity in exposure to mobile-source $PM_{2.5}$ for residents of overburdened communities.

This figure accompanies Fig. 3 (disparity for Hispanic Californians) by illustrating the disparity in exposure for residents of the two classifications of overburdened communities. The disparity in exposure for residents of overburdened communities as defined in AB617 (lefthand column) and SB535 (righthand column) is broken down in three ways to disaggregate contributions from LDVs, MDVs, HDVs, and all other vehicles following the same methodology as Fig. 3 and Fig. S9. The fractional contributions to disparity from each fleet type for AB617 and SB535 community members (A-D) on aggregate closely mirror that for Hispanic and Black Californians. However, the relative disparity from each mobile source is slightly different for AB617 community members (E), as they are most disparately exposed to $PM_{2.5}$ resulting from emissions from LDVs.

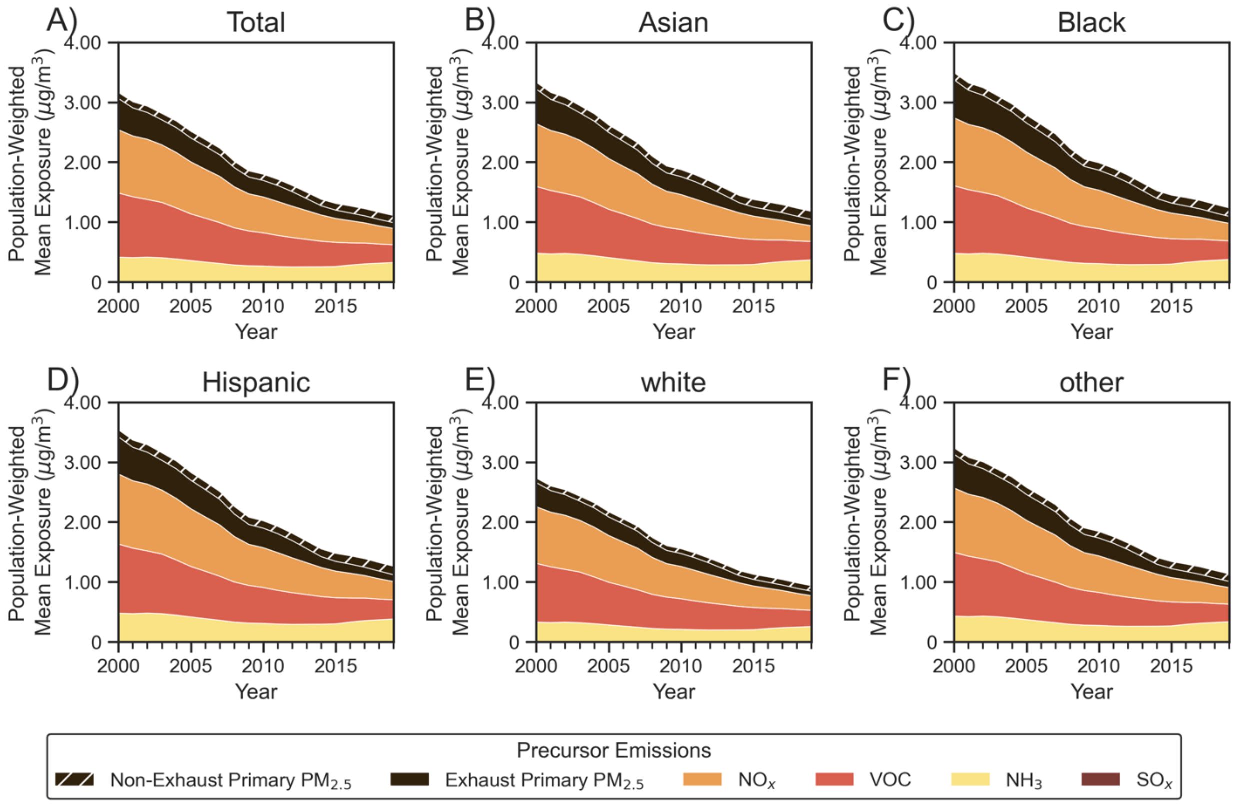

Fig. S11. Population-weighted mean exposure by precursor from the full mobile fleet. Contribution to total $PM_{2.5}$ concentration from emissions of each precursor pollutant from the full vehicle fleet across time by racial-ethnic group. Here, we perform a similar analysis to Fig. 3 of disaggregating results, but we show concentration from each individual precursor pollutant rather than fleet type. Contributions from primary $PM_{2.5}$ emissions are split by emissions mode, using hatching to represent non-exhaust (brake wear, tire wear) $PM_{2.5}$ emissions. The shape and contribution of each precursor does not change substantially between the statewide total population (A) and each racial-ethnic group (B-F). The relative contribution of each species is also roughly even across time and racial-ethnic group.

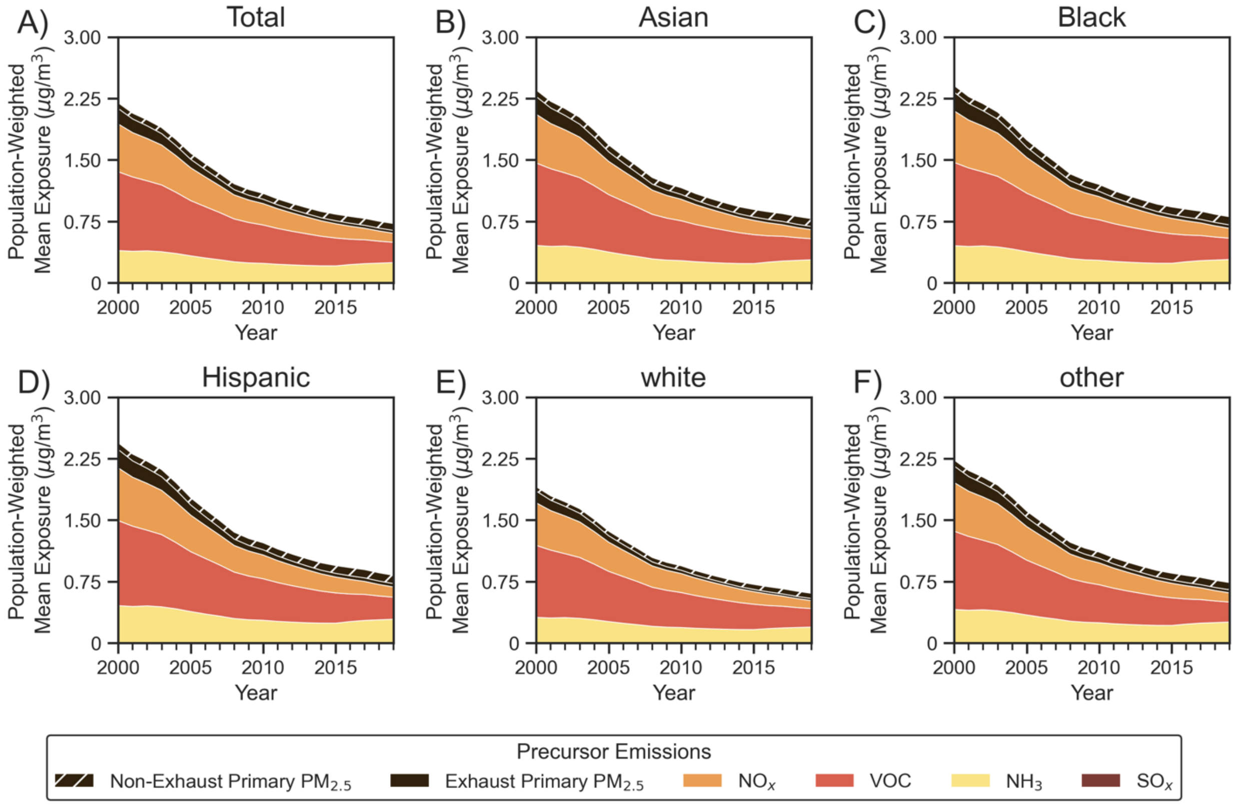

Fig. S12. Population-weighted mean exposure by precursor from the LDV fleet.
Contribution to total $PM_{2.5}$ concentration from emissions of each precursor pollutant from the LDV fleet across time by racial-ethnic group. Here, we repeat the analysis from Fig. S11 but for the LDV fleet alone. Again, the shape and contribution of each precursor does not change substantially between the statewide total population (A) and each racial-ethnic group (B-F). From the LDV fleet, emissions of NO_x, VOC, and NH_3 are most important for population-weighted mean exposures to total $PM_{2.5}$. During the second half of the study, the importance of exhaust- and non-exhaust primary $PM_{2.5}$ switch, leading to a roughly constant overall contribution from primary $PM_{2.5}$.

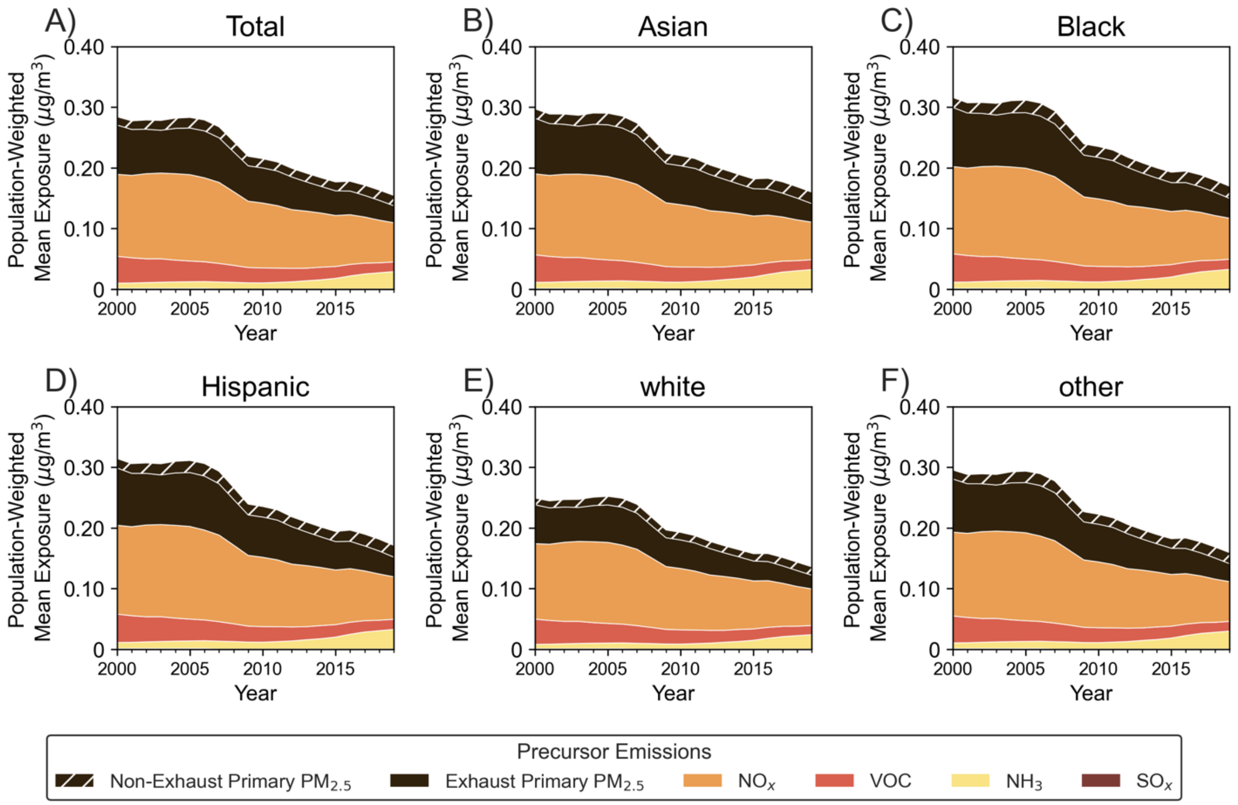

Fig. S13. Population-weighted mean exposure by precursor from the MDV fleet.

Contribution to total $PM_{2.5}$ concentration from emissions of each precursor pollutant from the MDV fleet across time by racial-ethnic group. Here, we repeat the analysis from Fig. S11 but for the MDV fleet alone. Again, the shape and contribution of each precursor does not change substantially between the statewide total population (A) and each racial-ethnic group (B-F). From the MDV fleet, emissions of primary $PM_{2.5}$ and NO_x are most important for population-weighted mean exposures to total $PM_{2.5}$. While relatively less important, contributions from NH_3 are increasing in the second half of the study, while contributions of VOC are declining.

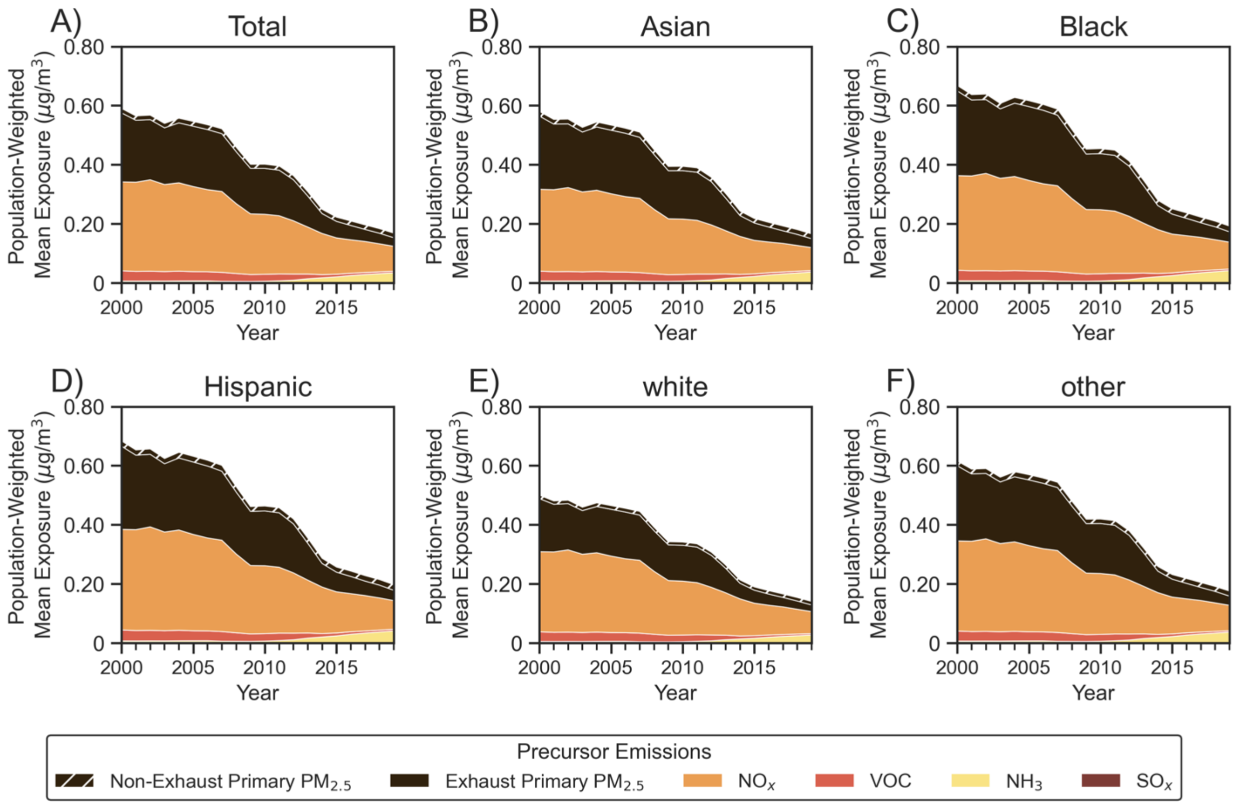

Fig. S14. Population-weighted mean exposure by precursor from the HDV fleet.
Contribution to total $PM_{2.5}$ concentration from emissions of each precursor pollutant from the HDV fleet across time by racial-ethnic group. Here, we repeat the analysis from Fig. S11 but for the HDV fleet alone. Again, the shape and contribution of each precursor does not change substantially between the statewide total population (A) and each racial/ethnic group (B-F). From the HDV fleet, emissions of primary $PM_{2.5}$ and NO_x are far more important for population-weighted mean exposures to total $PM_{2.5}$. During the second half of the study, the importance of VOC and NH_3 switch, with NH_3 becoming increasingly important for total $PM_{2.5}$ formation from HDVs. Similarly, while still important for HDV-derived $PM_{2.5}$, contributions from exhaust-mode primary $PM_{2.5}$ have reduced, resulting in a higher fractional contribution from NO_x.

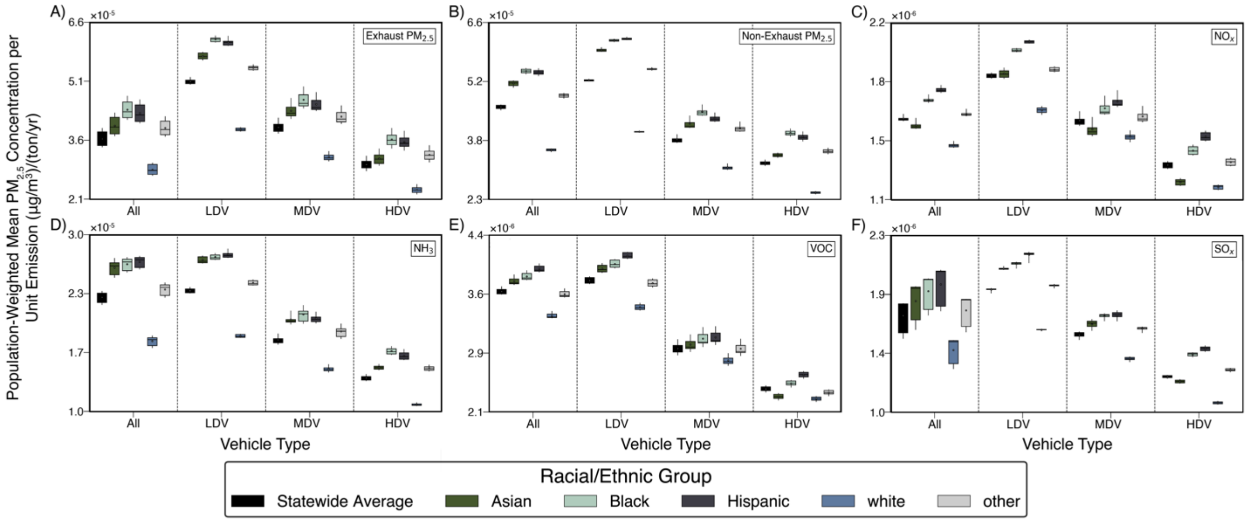

Fig. S15. Population-weighted mean exposure per unit emission for each fleet and racial-ethnic group.

We compare the per-unit impact of emissions of each precursor species from each vehicle type on population-weighted mean (PWM) exposure concentration for each race-ethnicity. Differences in this metric arise principally because of source patterns of proximity (i.e., on average, which vehicle types drive nearest to each group of people). This metric also depends on patterns of atmospheric transport and chemistry, but these patterns do not have a strong relationship with demographics. The box plots represent the distribution of this metric across the 19 years. The median is represented by the central bar, the mean is shown as a circle, the upper and lower quartiles are represented by the box, and the whiskers show the 10th and 90th percentile. For each vehicle type, the PWM concentration per unit emissions experienced by Black and Hispanic Californians is substantially higher than that experienced by white Californians, reflecting in large part the differences in proximity to vehicle activity. We see especially large exposures per unit of emissions of LDV emissions because LDV emissions tend to be ubiquitously concentrated in residential areas. In broad contours, these results are very similar for each of the emitted species.

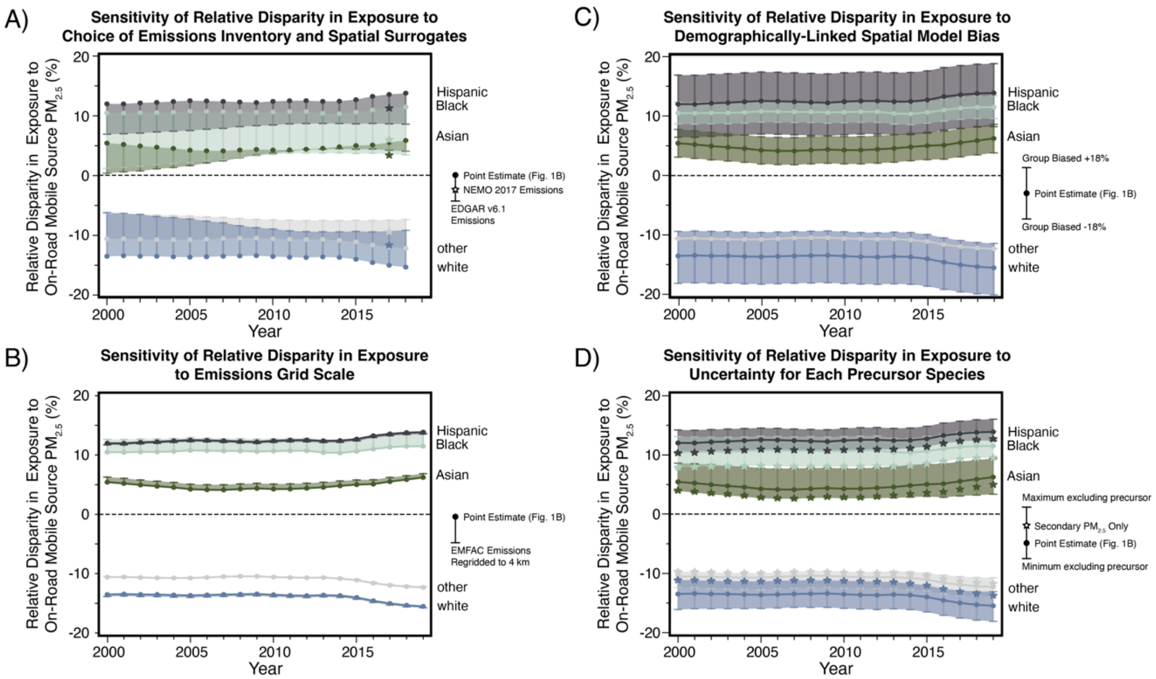

Fig. S16. Sensitivity analyses demonstrate our finding of persistent relative disparities over time is robust to a wide range of potential biases and uncertainties in emissions inventory and model performance.

Here, Fig. 1B is reproduced with error bars that show the sensitivity to four perturbations. The sensitivity tests are described in detail in the "Model Uncertainty and Sensitivity" subsection of the Supplementary Text. In Fig. S16A, we estimate relative disparity in exposure to emissions from two independent emissions inventories. The one-directional error bars indicate the relative disparity in exposure to emissions from the EDGAR model (gridded at 0.1°, or ~10 km); the relative disparity in exposure to emissions from the NEMO model (gridded at 1 km), which are only available for 2017, are indicated by a star. Regardless of the emissions model chosen, the relative disparities in exposure persist across time. In Fig. S16B, we show that our results are not influenced by error introduced by fine-scale spatial allocation. To do this, we re-grid the EMFAC emissions to a 2 km and 4 km grid. The error bars represent the relative disparity in exposure when emissions are gridded at 4 km and show minimal change in the magnitude or temporal pattern of disparity. In Fig. S16C, we perform a series of demographically linked perturbations to the modeled concentrations to simulate how spatial bias in the model could affect relative disparities in exposure. Error bars represent the range of relative disparities in exposure for each racial-ethnic group when concentrations are perturbed by ±18% in grid cells with 50% or more of the population. As shown in Fig. S16C, relative disparities in exposure persist, even if concentrations in predominantly Hispanic, Black, and Asian areas are overestimated and concentrations in predominantly white areas are underestimated. In Fig. S16D, we perform a systematic exclusion of each precursor pollutant and confirm that our results are robust to any model error associated with an individual precursor. The scenario where the excluded precursor is primary $PM_{2.5}$ (i.e., all $PM_{2.5}$ is secondary) is denoted with a star and represents the smallest relative disparity for all groups.

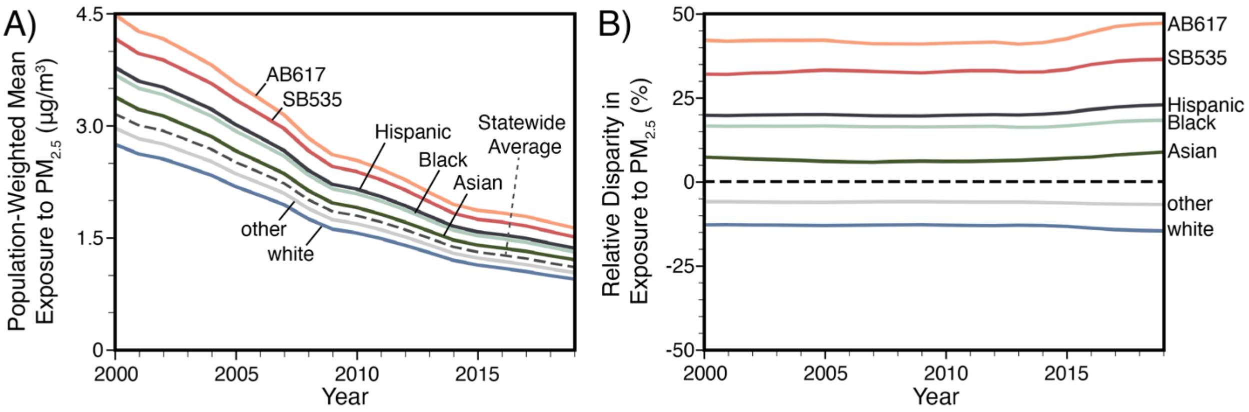

Fig. S17. On-road mobile-source $PM_{2.5}$ exposure and relative disparity in exposure for each demographic group using static 2000 population data.

Here, Fig. 1 is recreated using a static 2000 Census population instead of the static 2010 dataset used throughout this manuscript to evaluate the sensitivity of our finding to the population data. The main findings previously described have not changed: even with a static 2000 population, the exposure concentrations have decreased for all racial-ethnic groups (A) but the relative disparity in exposures have persisted (B). A key difference is that the overall population-weighted mean exposure concentration when using 2000 Census data is slightly higher for Californians of color, resulting in a higher relative disparity in exposure. This finding may suggest a population migration of people of color towards lower traffic areas between the 2000 and 2010 Census. Assuming the trend of population migration continued towards 2020, we might expect that the PWM exposure concentrations for each group might decrease further towards 2019. However, the persistence of the relative exposure disparity regardless of population dataset year suggests that the spatial distribution of emissions is not markedly variable over time, thereby leading to relatively constant relative disparities over time.

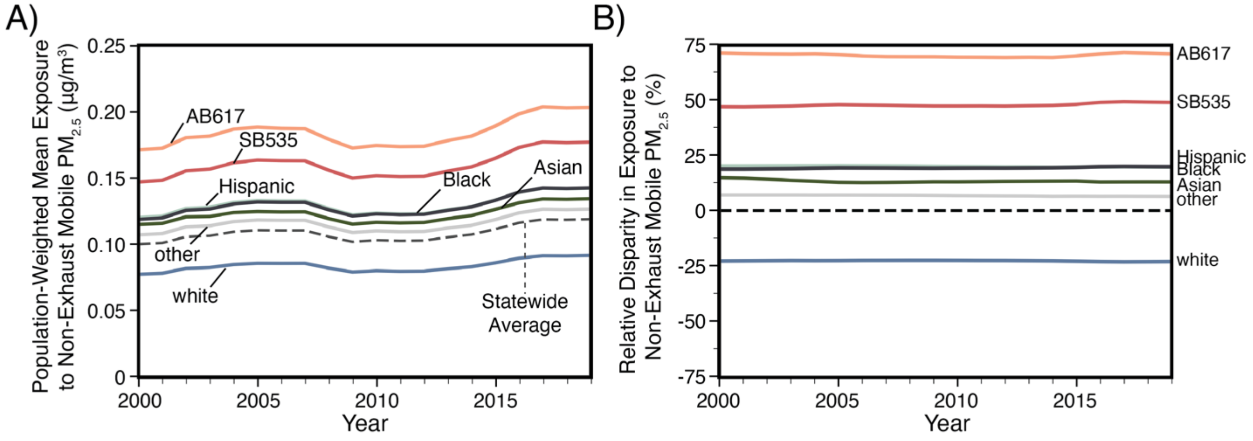

Fig. S18. Exposure and relative disparity in exposure from non-exhaust mobile-source $PM_{2.5}$ emissions for each demographic group.

Statewide population-weighted mean exposure concentrations (A) and relative disparity in exposure (B) to emissions of only non-exhaust $PM_{2.5}$ from on-road mobile sources during the study period. The Asian, Black, white, and other racial groups do not include those identifying as Hispanic. Here, we show that the population-weighted mean exposure to emissions of non-exhaust $PM_{2.5}$ has increased for all groups yet the relative disparity in exposure has remained constant. Notably, although the exposure concentration from non-exhaust sources is only approximately 1% of the current National Ambient Air Quality Standard, the magnitude of the relative disparity in exposure for all groups is larger for non-exhaust $PM_{2.5}$ than for the full set of emissions shown in Fig. 1. While this is not equivalent to modeling the future all-electric fleet, we can use the non-exhaust emissions from the full vehicle fleet as an analog for current driving patterns and emissions that might be relevant to disparities in an all-electric future. This sensitivity analysis suggests that relative disparities may persist even in the "all-electric future" where all vehicles across the fleet have zero exhaust emissions.

Table S1.
Vehicle fleet classifications for EMFAC2021 vehicle types used in this study.

EMFAC2021 Vehicle Type	Description from EMFAC2021[a]	GVWR (lb)[b]	Fleet
All Other Buses	All Other Buses	--	Other
LDA	Passenger Cars	--	LDV
LDT1	Light-Duty Trucks (ETW ≤ 3,750 lb)	< 6,000	LDV
LDT2	Light-Duty Trucks (ETW 3,751-5,750 lb)	< 6,000	LDV
LHD1	Light-Heavy-Duty Trucks	8,501-10,000	MDV
LHD2	Light-Heavy-Duty Trucks	10,001-14,000	MDV
MCY	Motorcycles	--	Other
MDV	Medium-Duty Trucks	5,751-8,500	LDV
MH	Motor Homes	--	Other
Motor Coach	Motor Coach	--	Other
OBUS	Other Buses	--	Other
PTO	Power Take Off	--	HDV
SBUS	School Buses	--	Other
T6 CAIRP Class 4	Medium-Heavy Duty CA International Registration Plan Truck	14,001-16,000	MDV
T6 CAIRP Class 5		16,001-19,500	MDV
T6 CAIRP Class 6		19,501-26,000	MDV
T6 CAIRP Class 7		26,001-33,000	MDV
T6 Instate Delivery Class 4	Medium-Heavy Duty Delivery Truck	14,001-16,000	MDV
T6 Instate Delivery Class 5		16,001-19,500	MDV
T6 Instate Delivery Class 6		19,501-26,000	MDV
T6 Instate Delivery Class 7		26,001-33,000	MDV
T6 Instate Other Class 4	Medium-Heavy Duty Other Truck	14,001-16,000	MDV
T6 Instate Other Class 5		16,001-19,500	MDV
T6 Instate Other Class 6		19,501-26,000	MDV
T6 Instate Other Class 7		26,001-33,000	MDV
T6 Instate Tractor Class 6	Medium-Heavy Duty Tractor Truck	19,501-26,000	MDV
T6 Instate Tractor Class 7		26,001-33,000	MDV
T6 OOS Class 4	Medium-Heavy Duty Out-Of-State Truck	14,001-16,000	MDV
T6 OOS Class 5		16,001-19,500	MDV
T6 OOS Class 6		19,501-26,000	MDV
T6 OOS Class 7		26,001-33,000	MDV
T6 Public Class 4	Medium-Heavy Duty Public Fleet Truck	14,001-16,000	MDV
T6 Public Class 5		16,001-19,500	MDV
T6 Public Class 6		19,501-26,000	MDV
T6 Public Class 7		26,001-33,000	MDV
T6 Utility Class 5	Medium-Heavy Duty Utility Fleet Truck	16,001-19,500	MDV
T6 Utility Class 6		19,501-26,000	MDV
T6 Utility Class 7		26,001-33,000	MDV
T6TS	Medium-Heavy Duty Truck	--	MDV
T7 CAIRP Class 8	Heavy-Heavy Duty CA International Registration Plan Truck	≥ 33,001	HDV
T7 NNOOS Class 8	Heavy-Heavy Duty Non-Neighboring Out-Of-State Truck	≥ 33,001	HDV

EMFAC2021 Vehicle Type	Description from EMFAC2021[a]	GVWR (lb)[b]	Fleet
T7 NOOS Class 8	Heavy-Heavy Duty Neighboring Out-Of-State Truck	≥ 33,001	HDV
T7 Other Port Class 8	Heavy-Heavy Duty Drayage Truck at Other Facilities	≥ 33,001	HDV
T7 POAK Class 8	Heavy-Heavy Duty Drayage Truck in Bay Area	≥ 33,001	HDV
T7 POLA Class 8	Heavy-Heavy Duty Drayage Truck near South Coast	≥ 33,001	HDV
T7 Public Class 8	Heavy-Heavy Duty Public Fleet Truck	≥ 33,001	HDV
T7 Single Concrete/Transit Mix Class 8	Heavy-Heavy Duty Single Unit Concrete/Transit Mix Truck	≥ 33,001	HDV
T7 Single Dump Class 8	Heavy-Heavy Duty Single Unit Dump Truck	≥ 33,001	HDV
T7 Single Other Class 8	Heavy-Heavy Duty Single Unit Other Truck	≥ 33,001	HDV
T7 SWCV Class 8	Heavy-Heavy Duty Solid Waste Collection Truck	≥ 33,001	HDV
T7 Tractor Class 8	Heavy-Heavy Duty Tractor Truck	≥ 33,001	HDV
T7 Utility Class 8	Heavy-Heavy Duty Utility Fleet Truck	≥ 33,001	HDV
T7IS	Heavy-Heavy Duty Truck	--	HDV
UBUS	Urban Buses	--	BUS

Notes:

a. Descriptions and GVWR are from Table 6.1 in the EMFAC2021 Volume III Technical Document, Version 1.0.0.

b. GVWR is not provided for all vehicle types.

Abbreviations:

GVWR = gross vehicle weight rating
ETW = emission test weight

Table S2.
California's population breakdown by race and ethnicity.

Racial/Ethnic Group [b]	Population Breakdown (%)				
	Statewide	AB617 Community [c]	Non-AB617 Community [c]	SB535 Community [d]	Non-SB535 Community [d]
Asian	12.8	7.5	13.3	8.8	14.5
Black	5.8	17.5	4.8	8.9	4.6
Hispanic	37.6	65.5	35.3	62.7	27.4
white	40.1	7.2	42.9	17.1	49.6
other	3.6	2.4	3.7	2.5	4.1

Notes:
[a.] Population data is queried from the 2010 Census at the Census tract level.
[b.] The Asian, Black, white, and other groups include only people who do not identify as Hispanic. The Hispanic group includes members of all races who identify as Hispanic.
[c.] Population by race and ethnicity at the tract level was apportioned to community boundaries defined by the California Air Resource Board using area allocation.
[d.] Population by race and ethnicity at the tract level was filtered to those tracts included in SB535.

Table S3.
Exposure and disparity to on-road vehicles by race-ethnicity.

Year	Racial-Ethnic Group					
	**Total**	**Asian**	**Black**	**Hispanic**	**white**	**other**
Population-Weighted Mean Exposure [a] (μg/m^3)						
2000	3.2	3.3	3.5	3.5	2.7	2.8
2001	3.0	3.2	3.3	3.4	2.6	2.7
2002	2.9	3.1	3.2	3.3	2.5	2.6
2003	2.8	2.9	3.1	3.2	2.4	2.5
2004	2.7	2.8	3.0	3.0	2.3	2.4
2005	2.5	2.6	2.8	2.8	2.2	2.2
2006	2.4	2.5	2.6	2.7	2.1	2.1
2007	2.2	2.3	2.5	2.5	1.9	2.0
2008	2.0	2.1	2.2	2.3	1.7	1.8
2009	1.9	1.9	2.0	2.1	1.6	1.7
2010	1.8	1.9	2.0	2.0	1.6	1.6
2011	1.7	1.8	1.9	1.9	1.5	1.5
2012	1.6	1.7	1.8	1.8	1.4	1.4
2013	1.5	1.6	1.7	1.7	1.3	1.3
2014	1.4	1.4	1.5	1.5	1.2	1.2
2015	1.3	1.4	1.4	1.5	1.1	1.2
2016	1.3	1.3	1.4	1.4	1.1	1.1
2017	1.2	1.3	1.4	1.4	1.0	1.1
2018	1.2	1.2	1.3	1.3	1.0	1.0
2019	1.1	1.2	1.2	1.3	0.9	1.0
Absolute Disparity [b, c] (μg/m^3)						
2000	--	0.2	0.3	0.4	-0.4	-0.3
2001	--	0.2	0.3	0.4	-0.4	-0.3
2002	--	0.1	0.3	0.4	-0.4	-0.3
2003	--	0.1	0.3	0.3	-0.4	-0.3
2004	--	0.1	0.3	0.3	-0.4	-0.3
2005	--	0.1	0.3	0.3	-0.3	-0.3
2006	--	0.1	0.3	0.3	-0.3	-0.3
2007	--	0.1	0.2	0.3	-0.3	-0.2

Year	Racial-Ethnic Group					
	Total	Asian	Black	Hispanic	white	other
Absolute Disparity [b, c] (µg/m^3)						
2008	--	0.1	0.2	0.2	-0.3	-0.2
2009	--	0.1	0.2	0.2	-0.2	-0.2
2010	--	0.1	0.2	0.2	-0.2	-0.2
2011	--	0.1	0.2	0.2	-0.2	-0.2
2012	--	0.1	0.2	0.2	-0.2	-0.2
2013	--	0.1	0.2	0.2	-0.2	-0.2
2014	--	0.1	0.1	0.2	-0.2	-0.1
2015	--	0.1	0.1	0.2	-0.2	-0.1
2016	--	0.1	0.1	0.2	-0.2	-0.1
2017	--	0.1	0.1	0.2	-0.2	-0.1
2018	--	0.1	0.1	0.2	-0.2	-0.1
2019	--	0.1	0.1	0.2	-0.2	-0.1
Relative Disparity [b, d] (%)						
2000	--	5.4	10.4	11.8	-13.4	-10.5
2001	--	5.2	10.5	12	-13.5	-10.6
2002	--	5	10.6	12.3	-13.7	-10.8
2003	--	4.7	10.6	12.3	-13.6	-10.8
2004	--	4.5	10.5	12.3	-13.5	-10.7
2005	--	4.3	10.8	12.6	-13.7	-10.8
2006	--	4.1	10.6	12.3	-13.5	-10.5
2007	--	4.2	10.8	12.6	-13.7	-10.7
2008	--	4.3	10.7	12.4	-13.6	-10.5
2009	--	4.3	10.3	11.9	-13.1	-10.2
2010	--	4.2	10.6	12.4	-13.5	-10.6
2011	--	4.3	10.8	12.6	-13.8	-10.7
2012	--	4.4	10.8	12.6	-13.8	-10.8
2013	--	4.5	10.4	12.4	-13.6	-10.6
2014	--	4.7	10.2	12.2	-13.5	-10.7
2015	--	5.0	10.7	12.8	-14.1	-11.2
2016	--	5.0	10.7	12.9	-14.2	-11.3
2017	--	5.6	11.5	13.8	-15.3	-12.2

Year	Racial-Ethnic Group					
	**Total**	**Asian**	**Black**	**Hispanic**	**white**	**other**
Relative Disparity [b, d] (%)						
2018	--	5.7	11.1	13.3	-14.9	-11.8
2019	--	6.3	11.6	14.0	-15.6	-12.4

Notes:

[a.] The population-weighted mean exposure is estimated by multiplying the annual average $PM_{2.5}$ concentration by the population of the demographic group of interest within that grid cell, summing across all grid cells, and dividing by the total population:

$$PWM_k = \frac{\sum_{i=1}^{n} p_{i,k} \times C_i}{\sum_{i=1}^{n} p_{i,k}}$$

[b.] A positive number indicates a higher than statewide average exposure disparity. Numbers shown here may not subtract correctly due to rounding.

[c.] The absolute disparity for a racial/ethnic group is estimated as the statewide average exposure ("Total") subtracted from the group's exposure: $D_{A,k} = PWM_k - PWM_T$

[d.] The relative disparity for a racial/ethnic group is the absolute disparity divided by the statewide average exposure concentration: $D_{R,k} = D_{A,k} / PWM_T$

Table S4.

Exposure to $PM_{2.5}$ from on-road vehicles by AB617 community.

Year	Population-Weighted Mean Exposure [a] (μg/m^3) by Community [b]									
	All [c]	*AL*	*BVHP*	*CECH*	*ECV*	*ELABHC*	*EOAK*	*IBC*	*IP1*	*RCHM*
2000	4.4	1.1	2.3	1.0	1.3	8.4	2.7	1.4	1.1	1.5
2001	4.2	1.1	2.2	0.9	1.3	7.9	2.5	1.4	1.0	1.4
2002	4.1	1.1	2.1	0.9	1.2	7.8	2.4	1.3	1.0	1.4
2003	3.9	1.0	2.0	0.9	1.2	7.5	2.3	1.3	1.0	1.3
2004	3.8	1.0	1.8	0.8	1.2	7.2	2.2	1.2	0.9	1.2
2005	3.5	1.0	1.7	0.8	1.1	6.7	2.1	1.1	0.9	1.1
2006	3.3	1.0	1.6	0.7	1.1	6.3	2.0	1.1	0.8	1.1
2007	3.1	0.9	1.5	0.7	1.0	5.9	1.9	1.0	0.8	1.0
2008	2.8	0.8	1.4	0.6	0.9	5.3	1.7	0.9	0.7	0.9
2009	2.6	0.8	1.3	0.6	0.8	4.9	1.5	0.8	0.6	0.9
2010	2.5	0.7	1.2	0.6	0.8	4.8	1.5	0.8	0.6	0.8
2011	2.4	0.7	1.1	0.5	0.8	4.5	1.4	0.8	0.6	0.8
2012	2.2	0.6	1.1	0.5	0.7	4.3	1.3	0.7	0.5	0.7
2013	2.1	0.6	1.0	0.4	0.7	4.0	1.2	0.7	0.5	0.7
2014	1.9	0.5	1.0	0.4	0.6	3.7	1.1	0.6	0.4	0.6
2015	1.8	0.5	0.9	0.4	0.6	3.5	1.1	0.6	0.4	0.6
2016	1.8	0.4	0.9	0.4	0.5	3.5	1.0	0.6	0.4	0.6
2017	1.8	0.4	0.9	0.3	0.5	3.5	1.0	0.5	0.4	0.5
2018	1.7	0.4	0.9	0.3	0.5	3.3	0.9	0.5	0.3	0.5
2019	1.6	0.3	0.9	0.3	0.5	3.2	0.9	0.5	0.3	0.5
	SBM	*SCFR*	*SDPC*	*SELA*	*SHFT*	*SLA*	*SSACF*	*STCK*	*WOAK*	*WWLBC*
2000	4.1	2.7	3.5	6.1	1.3	6.1	2.2	2.2	2.7	3.8
2001	3.9	2.5	3.3	5.8	1.2	5.8	2.1	2.1	2.5	3.6
2002	3.8	2.5	3.3	5.6	1.2	5.7	2.1	2.0	2.4	3.5
2003	3.7	2.4	3.1	5.4	1.2	5.4	2.0	1.9	2.3	3.4
2004	3.6	2.4	3.0	5.2	1.2	5.2	1.9	1.9	2.2	3.2
2005	3.4	2.3	2.8	4.8	1.1	4.8	1.8	1.8	2.1	3.0
2006	3.2	2.2	2.6	4.5	1.1	4.5	1.8	1.7	2.0	2.8
2007	3.0	2.1	2.5	4.2	1.0	4.2	1.7	1.6	1.9	2.6
2008	2.7	1.9	2.3	3.8	0.9	3.8	1.5	1.5	1.7	2.4
2009	2.5	1.7	2.1	3.5	0.8	3.5	1.4	1.3	1.6	2.2

Year	Population-Weighted Mean Exposure [a] (μg/m^3) by Community [b]									
	SBM	*SCFR*	*SDPC*	*SELA*	*SHFT*	*SLA*	*SSACF*	*STCK*	*WOAK*	*WWLBC*
2010	2.4	1.7	2.0	3.4	0.8	3.4	1.3	1.3	1.5	2.1
2011	2.3	1.6	1.9	3.2	0.8	3.3	1.3	1.2	1.5	2.0
2012	2.2	1.5	1.8	3.1	0.7	3.1	1.2	1.2	1.4	1.9
2013	2.0	1.4	1.7	2.8	0.6	2.8	1.1	1.1	1.3	1.8
2014	1.9	1.2	1.5	2.6	0.6	2.6	1.0	1.0	1.1	1.6
2015	1.8	1.1	1.4	2.5	0.5	2.5	0.9	0.9	1.1	1.6
2016	1.7	1.0	1.4	2.5	0.5	2.5	0.9	0.8	1.0	1.5
2017	1.7	0.9	1.3	2.4	0.4	2.5	0.8	0.8	1.0	1.5
2018	1.6	0.9	1.3	2.3	0.4	2.4	0.8	0.7	1.0	1.4
2019	1.5	0.8	1.3	2.2	0.4	2.3	0.7	0.7	0.9	1.4

Notes:

a. The population-weighted mean exposure is estimated by multiplying the annual average $PM_{2.5}$ concentration by the population of the demographic group of interest within that grid cell, summing across all grid cells, and dividing by the total population:

$$PWM_k = \frac{\sum_{i=1}^{n} P_{i,k} \times C_i}{\sum_{i=1}^{n} P_{i,k}}$$

b. The communities are identified using abbreviated codes. These codes are as follows:

- AL = Arvin, Lamont
- BVHP = Bayview Hunters Point/Southeast San Francisco
- CECH = El Centro, Heber, Calexico
- ECV = Eastern Coachella Valley
- ELABHC = East Los Angeles, Boyle Heights, West Commerce
- EOAK = East Oakland
- IBC = International Border Community
- IP1 = Northern Imperial Phase 1
- RCHM = Richmond – San Pablo
- SBM = San Bernardino, Muscoy
- SCFR = South Central Fresno
- SDPC = Portside Environmental Justice Neighborhoods
- SELA = South East Los Angeles
- SHFT = Shafter
- SLA = South Los Angeles
- SSACF = South Sacramento - Florin
- STCK = Stockton
- WOAK = West Oakland
- WWLBC = Wilmington, Carson, West Long Beach

c. The “All” column refers to the combined population-weighted mean exposure across all communities.

Table S5.
Exposure and disparity to on-road vehicles by income quartile.

Year	Income Quartile [a]				
	**Total**	**Q1**	**Q2**	**Q3**	**Q4**
Population-Weighted Mean Exposure [b] (µg/m^3)					
2000	3.2	3.5	3.2	3.0	2.9
2001	3.0	3.3	3.1	2.9	2.8
2002	2.9	3.2	3.0	2.8	2.7
2003	2.8	3.1	2.9	2.7	2.6
2004	2.7	3.0	2.7	2.6	2.4
2005	2.5	2.8	2.6	2.4	2.3
2006	2.4	2.7	2.4	2.3	2.1
2007	2.2	2.5	2.3	2.1	2.0
2008	2.0	2.2	2.1	1.9	1.8
2009	1.9	2.1	1.9	1.8	1.7
2010	1.8	2.0	1.8	1.7	1.6
2011	1.7	1.9	1.8	1.6	1.5
2012	1.6	1.8	1.6	1.5	1.5
2013	1.5	1.7	1.5	1.4	1.4
2014	1.4	1.5	1.4	1.3	1.2
2015	1.3	1.5	1.3	1.3	1.2
2016	1.3	1.4	1.3	1.2	1.1
2017	1.2	1.4	1.3	1.2	1.1
2018	1.2	1.3	1.2	1.1	1.0
2019	1.1	1.2	1.1	1.1	1.0
Absolute Disparity [c, d] (µg/m^3)					
2000	--	0.3	0.1	-0.1	-0.3
2001	--	0.3	0.1	-0.1	-0.3
2002	--	0.3	0.1	-0.1	-0.3
2003	--	0.3	0.1	-0.1	-0.3
2004	--	0.3	0.1	-0.1	-0.3
2005	--	0.3	0.1	-0.1	-0.2
2006	--	0.3	0.1	-0.1	-0.2
2007	--	0.3	0.1	-0.1	-0.2

Year	Income Quartile [a]				
	**Total**	**Q1**	**Q2**	**Q3**	**Q4**
Absolute Disparity [c, d] (μg/m³)					
2008	--	0.2	0.0	-0.1	-0.2
2009	--	0.2	0.0	-0.1	-0.2
2010	--	0.2	0.0	-0.1	-0.2
2011	--	0.2	0.0	-0.1	-0.2
2012	--	0.2	0.0	-0.1	-0.2
2013	--	0.2	0.0	-0.1	-0.1
2014	--	0.2	0.0	-0.1	-0.1
2015	--	0.1	0.0	-0.1	-0.1
2016	--	0.1	0.0	-0.1	-0.1
2017	--	0.1	0.0	-0.1	-0.1
2018	--	0.1	0.0	0.0	-0.1
2019	--	0.1	0.0	0.0	-0.1
Relative Disparity [c, e] (%)					
2000	--	10.3	1.9	-3.7	-8.4
2001	--	10.4	1.9	-3.7	-8.5
2002	--	10.7	2.0	-3.8	-8.8
2003	--	10.8	2.1	-3.8	-9.0
2004	--	11.2	2.2	-3.9	-9.3
2005	--	11.6	2.3	-4.0	-9.7
2006	--	11.6	2.3	-4.1	-9.7
2007	--	11.6	2.3	-4.1	-9.7
2008	--	11.5	2.2	-4.0	-9.6
2009	--	11.3	2.2	-4.0	-9.5
2010	--	11.5	2.3	-4.0	-9.7
2011	--	11.6	2.3	-4.0	-9.8
2012	--	11.5	2.3	-4.0	-9.8
2013	--	11.2	2.3	-3.9	-9.5
2014	--	10.9	2.3	-3.8	-9.4
2015	--	11.1	2.4	-3.9	-9.6
2016	--	11.8	2.7	-4.0	-10.3
2017	--	12.1	2.7	-4.1	-10.6

Year	Income Quartile [a]				
	Total	Q1	Q2	Q3	Q4
Relative Disparity [c, e] (%)					
2018	--	12.2	2.8	-4.1	-10.8
2019	--	12.1	2.9	-4.1	-10.8

Notes:

[a.] Income quartiles are defined as follows. Total population and median household income (adjusted to 2012 inflation) were queried at the block group level from the 2008-2012 American Community Survey. Income data were adjusted to 2010 using the Consumer Price Index. Block groups were then sorted by median income level and the percentile of the overall population was estimated as the cumulative number of people at that income level divided by the total population: < $40,892 Q1, $40,893-$58,452 Q2, $58,453-$82,842 Q3, and >$82,843 Q4. Each quartile represents approximately 9.3 million Californians.

[b.] The population-weighted mean exposure is estimated by multiplying the annual average $PM_{2.5}$ concentration by the population of the demographic group of interest within that grid cell, summing across all grid cells, and dividing by the total population: $PWM_k = \frac{\sum_{i=1}^{n} P_{i,k} \times C_i}{\sum_{i=1}^{n} P_{i,k}}$

[c.] A positive number indicates a higher than statewide average exposure disparity. Numbers shown here may not subtract correctly due to rounding.

[d.] The absolute disparity for an income group is estimated as the statewide average exposure ("Total") subtracted from the group's exposure:
$D_{A,k} = PWM_k - PWM_T$

[e.] The relative disparity for an income group is the absolute disparity divided by the statewide average exposure concentration.
$D_{R,k} = D_{A,k} / PWM_T$

Table S6.
Population-weighted mean exposure by demographic group and vehicle fleet.

Year	Population-Weighted Mean Exposure [a] by Demographic Group							
	Total	Asian	Black	Hispanic	white	other	AB617 [b]	SB535 [b]
Full Fleet								
2000	3.2	3.3	3.5	3.5	2.7	2.8	4.4	4.1
2001	3.0	3.2	3.3	3.4	2.6	2.7	4.2	3.9
2002	2.9	3.1	3.2	3.3	2.5	2.6	4.1	3.8
2003	2.8	2.9	3.1	3.2	2.4	2.5	3.9	3.6
2004	2.7	2.8	3.0	3.0	2.3	2.4	3.8	3.5
2005	2.5	2.6	2.8	2.8	2.2	2.2	3.5	3.3
2006	2.4	2.5	2.6	2.7	2.1	2.1	3.3	3.1
2007	2.2	2.3	2.5	2.5	1.9	2.0	3.1	2.9
2008	2.0	2.1	2.2	2.3	1.7	1.8	2.8	2.6
2009	1.9	1.9	2.0	2.1	1.6	1.7	2.6	2.4
2010	1.8	1.9	2.0	2.0	1.6	1.6	2.5	2.3
2011	1.7	1.8	1.9	1.9	1.5	1.5	2.4	2.2
2012	1.6	1.7	1.8	1.8	1.4	1.4	2.2	2.1
2013	1.5	1.6	1.7	1.7	1.3	1.3	2.1	1.9
2014	1.4	1.4	1.5	1.5	1.2	1.2	1.9	1.8
2015	1.3	1.4	1.4	1.5	1.1	1.2	1.8	1.7
2016	1.3	1.3	1.4	1.4	1.1	1.1	1.8	1.7
2017	1.2	1.3	1.4	1.4	1.0	1.1	1.8	1.6
2018	1.2	1.2	1.3	1.3	1.0	1.0	1.7	1.5
2019	1.1	1.2	1.2	1.3	0.9	1.0	1.6	1.5
LDV Fleet								
2000	2.2	2.4	2.4	2.4	1.9	1.9	3.1	2.8
2001	2.1	2.2	2.3	2.3	1.8	1.8	2.9	2.6
2002	2.0	2.1	2.2	2.2	1.7	1.8	2.8	2.5
2003	1.9	2.0	2.1	2.1	1.6	1.7	2.7	2.4
2004	1.7	1.9	1.9	2.0	1.5	1.5	2.5	2.2
2005	1.6	1.7	1.7	1.8	1.4	1.4	2.3	2.0
2006	1.5	1.5	1.6	1.6	1.3	1.3	2.1	1.9
2007	1.3	1.4	1.5	1.5	1.2	1.2	1.9	1.7
2008	1.2	1.3	1.3	1.4	1.0	1.1	1.7	1.5

Year	Population-Weighted Mean Exposure [a] by Demographic Group							
	Total	Asian	Black	Hispanic	white	other	AB617 [b]	SB535 [b]
LDV Fleet								
2009	1.1	1.2	1.3	1.3	1.0	1.0	1.6	1.4
2010	1.1	1.2	1.2	1.2	0.9	1.0	1.6	1.4
2011	1.0	1.1	1.1	1.1	0.9	0.9	1.5	1.3
2012	1.0	1.0	1.1	1.1	0.8	0.9	1.4	1.2
2013	0.9	1.0	1.0	1.0	0.8	0.8	1.3	1.2
2014	0.9	0.9	1.0	1.0	0.7	0.8	1.2	1.1
2015	0.8	0.9	0.9	0.9	0.7	0.7	1.2	1.1
2016	0.8	0.9	0.9	0.9	0.7	0.7	1.2	1.1
2017	0.8	0.8	0.9	0.9	0.7	0.7	1.2	1.0
2018	0.8	0.8	0.8	0.9	0.6	0.6	1.1	1.0
2019	0.7	0.8	0.8	0.8	0.6	0.6	1.1	1.0
MDV Fleet								
2000	0.3	0.3	0.3	0.3	0.2	0.3	0.4	0.4
2001	0.3	0.3	0.3	0.3	0.2	0.3	0.4	0.4
2002	0.3	0.3	0.3	0.3	0.2	0.3	0.4	0.4
2003	0.3	0.3	0.3	0.3	0.2	0.3	0.4	0.3
2004	0.3	0.3	0.3	0.3	0.3	0.3	0.4	0.4
2005	0.3	0.3	0.3	0.3	0.3	0.3	0.4	0.4
2006	0.3	0.3	0.3	0.3	0.2	0.3	0.4	0.3
2007	0.3	0.3	0.3	0.3	0.2	0.3	0.3	0.3
2008	0.2	0.2	0.3	0.3	0.2	0.2	0.3	0.3
2009	0.2	0.2	0.2	0.2	0.2	0.2	0.3	0.3
2010	0.2	0.2	0.2	0.2	0.2	0.2	0.3	0.3
2011	0.2	0.2	0.2	0.2	0.2	0.2	0.3	0.3
2012	0.2	0.2	0.2	0.2	0.2	0.2	0.3	0.2
2013	0.2	0.2	0.2	0.2	0.2	0.2	0.2	0.2
2014	0.2	0.2	0.2	0.2	0.2	0.2	0.2	0.2
2015	0.2	0.2	0.2	0.2	0.2	0.2	0.2	0.2
2016	0.2	0.2	0.2	0.2	0.2	0.2	0.2	0.2
2017	0.2	0.2	0.2	0.2	0.2	0.2	0.2	0.2
2018	0.2	0.2	0.2	0.2	0.1	0.2	0.2	0.2
2019	0.2	0.2	0.2	0.2	0.1	0.1	0.2	0.2

Year	Population-Weighted Mean Exposure [a] by Demographic Group							
	Total	Asian	Black	Hispanic	white	other	AB617 [b]	SB535 [b]
HDV Fleet								
2000	0.6	0.6	0.7	0.7	0.5	0.5	0.8	0.8
2001	0.6	0.6	0.6	0.7	0.5	0.5	0.8	0.8
2002	0.6	0.6	0.6	0.7	0.5	0.5	0.8	0.8
2003	0.5	0.5	0.6	0.6	0.5	0.5	0.7	0.8
2004	0.6	0.5	0.6	0.6	0.5	0.5	0.8	0.8
2005	0.5	0.5	0.6	0.6	0.5	0.5	0.7	0.8
2006	0.5	0.5	0.6	0.6	0.5	0.5	0.7	0.8
2007	0.5	0.5	0.6	0.6	0.4	0.5	0.7	0.7
2008	0.5	0.5	0.5	0.5	0.4	0.4	0.6	0.6
2009	0.4	0.4	0.5	0.5	0.3	0.4	0.5	0.6
2010	0.4	0.4	0.5	0.5	0.3	0.4	0.5	0.6
2011	0.4	0.4	0.4	0.5	0.3	0.4	0.5	0.6
2012	0.4	0.4	0.4	0.4	0.3	0.3	0.5	0.5
2013	0.3	0.3	0.3	0.4	0.3	0.3	0.4	0.4
2014	0.2	0.2	0.3	0.3	0.2	0.2	0.3	0.3
2015	0.2	0.2	0.3	0.3	0.2	0.2	0.3	0.3
2016	0.2	0.2	0.2	0.2	0.2	0.2	0.3	0.3
2017	0.2	0.2	0.2	0.2	0.2	0.2	0.3	0.3
2018	0.2	0.2	0.2	0.2	0.2	0.2	0.3	0.3
2019	0.2	0.2	0.2	0.2	0.1	0.1	0.2	0.2

Notes:

a. The population-weighted mean exposure is estimated by multiplying the annual average $PM_{2.5}$ concentration by the population of the demographic group of interest within that grid cell, summing across all grid cells, and dividing by the total population:

$$PWM_k = \frac{\sum_{i=1}^{n} P_{i,k} \times C_i}{\sum_{i=1}^{n} P_{i,k}}$$

b. Members of overburdened communities as designated by California's AB617 and SB535 are included under their respective policy names.

REFERENCES AND NOTES

1. D. A. Paolella, C. W. Tessum, P. J. Adams, J. S. Apte, S. Chambliss, J. Hill, N. Z. Mueller, J. D. Marshall, Effect of model spatial resolution on estimates of fine particulate matter exposure and exposure disparities in the United States. *Environ. Sci. Technol. Lett.* **5**, 436–441 (2018).

2. C. W. Tessum, D. A. Paolella, S. E. Chambliss, J. S. Apte, J. D. Hill, J. D. Marshall, $PM_{2.5}$ polluters disproportionately and systemically affect people of color in the United States. *Sci. Adv.* **7**, eabf4491 (2021).

3. L. P. Clark, M. H. Harris, J. S. Apte, J. D. Marshall, National and intraurban air pollution exposure disparity estimates in the United States: Impact of data-aggregation spatial scale. *Environ. Sci. Technol. Lett.* **9**, 786–791 (2022).

4. H. M. Lane, R. Morello-Frosch, J. D. Marshall, J. S. Apte, Historical redlining is associated with present-day air pollution disparities in U.S. cities. *Environ. Sci. Technol. Lett.* **9**, 345–350 (2022).

5. T. W. Collins, S. E. Grineski, Y. Shaker, C. J. Mullen, Communities of color are disproportionately exposed to long-term and short-term $PM_{2.5}$ in metropolitan America. *Environ. Res.* **214**, 114038 (2022).

6. J. Colmer, I. Hardman, J. Shimshack, J. Voorheis, Disparities in $PM_{2.5}$ air pollution in the United States. *Science* **369**, 575–578 (2020).

7. J. Liu, J. D. Marshall, Spatial decomposition of air pollution concentrations highlights historical causes for current exposure disparities in the United States. *Environ. Sci. Technol. Lett.* **10**, 280–286 (2023).

8. C. W. Tessum, J. S. Apte, A. L. Goodkind, N. Z. Mueller, K. A. Mullins, D. A. Paolella, S. Polasky, N. P. Springer, S. K. Thakrar, J. D. Marshall, J. D. Hill, Inequity in consumption of goods and services adds to racial–ethnic disparities in air pollution exposure. *Proc. Natl. Acad. Sci. U.S.A.* **116**, 6001–6006 (2019).

9. Y. Wang, J. S. Apte, J. D. Hill, C. E. Ivey, R. F. Patterson, A. L. Robinson, C. W. Tessum, J. D. Marshall, Location-specific strategies for eliminating US national racial-ethnic $PM_{2.5}$ exposure inequality. *Proc. Natl. Acad. Sci. U.S.A.* **119**, e2205548119 (2022).

10. J. Liu, L. P. Clark, M. J. Bechle, A. Hajat, S. Y. Kim, A. L. Robinson, L. Sheppard, A. A. Szpiro, J. D. Marshall, Disparities in air pollution exposure in the United States by race/ethnicity and income, 1990–2010. *Environ. Health Perspect.* **129**, 127005 (2021).

11. A. Jbaily, X. Jhou, J. Liu, T.-H. Lee, L. Kamareddine, S. Verguet, F. Dominici, Air pollution exposure disparities across US population and income groups. *Nature* **601**, 228–233 (2022).

12. G. H. Kerr, A. van Donkelaar, R. V. Martin, M. Brauer, K. Bukart, S. Wozniak, D. L. Goldberg, S. C. Anenberg, Increasing racial and ethnic disparities in ambient air pollution-attributable morbidity and mortality in the United States. *Environ. Health Perspect.* **132**, 037002 (2024).

13. Y. Wen, S. Zhang, Y. Wang, J. Yang, L. He, Y. Wu, J. Hao, Dynamic traffic data in machine-learning air quality mapping improves environmental justice assessment. *Environ. Sci. Technol.* **58**, 3118–3128 (2024).

14. Q. Yu, B. Y. He, J. Ma, Y. Zhu, California's zero-emission vehicle adoption brings air quality benefits yet equity gaps persist. *Nat. Commun.* **14**, 7798 (2023).

15. J. S. Apte, S. E. Chambliss, C. W. Tessum, J. D. Marshall, "A method to prioritize sources for reducing high PM2.5 exposures in environmental justice communities in California" (Research Report, contract 17RD006, California Air Resources Board, 2019); https://ww2.arb.ca.gov/sites/default/files/classic/research/apr/past/17rd006.pdf.

16. S. E. Chambliss, C. P. R. Pinon, K. P. Messier, B. LaFranchi, C. R. Upperman, M. M. Lunden, A. L. Robinson, J. D. Marshall, J. S. Apte, Local- and regional-scale racial and ethnic disparities in air pollution determined by long-term mobile monitoring. *Proc. Natl. Acad. Sci. U.S.A.* **118**, e2109249118 (2021).

17. L. Cushing, D. Blaustein-Rejto, M. Wander, M. Pastor, J. Sadd, A. Zhu, R. Morello-Frosch, Carbon trading, co-pollutants, and environmental equity: Evidence from California's cap-and-trade program (2011–2015). *PLOS Med.* **15**, e1002604 (2018).

18. Y. Ju, L. J. Cushing, R. Morello-Frosch, An equity analysis of clean vehicle rebate programs in California. *Clim. Change* **162**, 2087–2105 (2020).

19. Y. Wang, J. S. Apte, J. D. Hill, C. E. Ivey, D. Johnson, E. Min, R. Morello-Frosch, R. Patterson, A. L. Robinson, C. W. Tessum, J. D. Marshall, Air quality policy should quantify effects on disparities. *Science* **381**, 272–274 (2023).

20. P. Picciano, M. Qiu, S. D. Eastham, M. Yuan, J. Reilly, N. E. Selin, Air quality related equity implications of U.S. decarbonization policy. *Nat. Commun.* **14**, 5543 (2023).

21. P. Polonik, K. Ricke, S. Reese, J. Burney, Air quality equity in US climate policy. *Proc. Natl. Acad. Sci. U.S.A.* **120**, e2217124120 (2023).

22. E. Spiller, J. Proville, A. Roy, N. Z. Muller, Mortality risk from $PM_{2.5}$: A comparison of modeling approaches to identify disparities across racial/ethnic groups in policy outcomes. *Environ. Health Perspect.* **129**, 127004 (2021).

23. S. F. Camilleri, A. Montgomery, M. A. Visa, J. L. Schnell, Z. E. Adelman, M. Janssen, E. A. Grubert, S. C. Anenberg, D. E. Horton, Air quality, health and equity implications of electrifying heavy-duty vehicles. *Nat. Sustain.* **6**, 1643–1653 (2023).

24. S. F. Camilleri, G. H. Kerr, S. C. Anenberg, D. E. Horton, All-Cause NO_2-attributable mortality burden and associated racial and ethnic disparities in the United States. *Environ. Sci. Technol. Lett.* **10**, 1159–1164 (2023).

25. M. A. Visa, S. F. Camilleri, A. Montgomery, J. L. Schnell, M. Janssen, Z. E. Adelman, S. C. Anenberg, E. A. Grubert, D. E. Horton, Neighborhood-scale air quality, public health, and equity implications of multi-modal vehicle electrification. *Environ. Res. Infrastruct. Sustain.* **3**, 035007 (2023).

26. V. A. Southerland, S. C. Anenberg, M. Harris, J. Apte, P. Hystad, A. van Donkelaar, R. V. Martin, M. Beyers, A. Roy, Assessing the distribution of air pollution health risks within cities: A neighborhood-scale analysis leveraging high-resolution data sets in the Bay Area, California. *Environ. Health Perspect.* **129**, 037006 (2021).

27. M. D. Castillo, P. L. Kinney, V. Southerland, C. A. Arno, K. Crawford, A. van Donkelaar, M. Hammer, R. V. Martin, S. C. Anenberg, Estimating intra-urban inequities in $PM_{2.5}$-attributable health impacts: A case study for Washington, DC. *Geohealth* **5**, e2021GH000431 (2021).

28. A. L. Goodkind, C. W. Tessum, J. S. Coggins, J. D. Hill, J. D. Marshall, Fine-scale damage estimates of particulate matter air pollution reveal opportunities for location-specific mitigation of emissions. *Proc. Natl. Acad. Sci. U.S.A.* **116**, 8775–8780 (2019).

29. G. Boeing, Y. Lu, C. Pilgram, Local inequities in the relative production of and exposure to vehicular air pollution in Los Angeles. *Urban Stud.* **60**, 2351–2368 (2023).

30. Y. Lu, Drive less but exposed more? Exploring social injustice in vehicular air pollution exposure. *Soc. Sci. Res.* **111**, 102867 (2023).

31. G. C. Pratt, M. L. Vadali, D. L. Kvale, K. M. Ellickson, Traffic, air pollution, minority and socio-economic status: Addressing inequities in exposure and risk. *Int. J. Environ. Res. Public Health* **12**, 5355–5372 (2015).

32. D. Houston, J. Wu, P. Ong, A. Winer, Structural disparities of urban traffic in Southern California: Implications for vehicle-related air pollution exposure in minority and high-poverty neighborhoods. *J. Urban Aff.* **26**, 565–592 (2004).

33. F. Garcia-Menendez, R. K. Saari, E. Monier, N. E. Selin, U.S. Air quality and health benefits from avoided climate change under greenhouse gas mitigation. *Environ. Sci. Technol.* **49**, 7580–7588 (2015).

34. California Air Resources Board, History (California Air Resources Board, 2023); https://ww2.arb.ca.gov/about/history.

35. United States Code of Federal Regulations. Clean Air Act Extension of 1970: Section 177. 42 CFR § 7507 (2) - New Motor Vehicle Emission Standards in Nonattainment Areas (1977); www.law.cornell.edu/uscode/text/42/7507.

36. C. Pappalardo, What a difference a state makes: California's authority to regulate motor vehicle emissions under the clean air act and the future of state autonomy. *Mich. J. Environ. Admin. Law* **10**, 169–223 (2021).

37. S. Samuelsen, S. Zhu, M. M. Kinnon, O. K. Yang, D. Dabdub, J. Brouwer, An episodic assessment of vehicle emission regulations on saving lives in California. *Environ. Sci. Technol.* **55**, 547–552 (2021).

38. California Air Resources Board, EMFAC2021 volume III technical document, version 1.0.1. California Air Resources Board (2021); https://ww2.arb.ca.gov/sites/default/files/2021-07/emfac2021_tech_doc_april2021.pdf.

39. California Air Resources Board, Estimated annual average emissions statewide (California Air Resources Board, 2023); https://ww2.arb.ca.gov/applications/statewide-emissions.

40. G. A. Ban-Weiss, J. P. McLaughlin, R. A. Harley, M. M. Lunden, T. W. Kirchstetter, A. J. Kean, A. W. Strawa, E. D. Stevenson, G. R. Kendall, Long-term changes in emissions of nitrogen oxides and particulate matter from on-road gasoline and diesel vehicles. *Atmos. Environ.* **42**, 220–232 (2008).

41. A. J. Kean, D. Littlejohn, G. A. Ban-Weiss, R. A. Harley, T. W. Kirchstetter, M. M. Lunden, Trends in on-road vehicle emissions of ammonia. *Atmos. Environ.* **43**, 1565–1570 (2009).

42. T. R. Dallmann, R. A. Harley, Evaluation of mobile source emission trends in the United States. *J. Geophys. Res. Atmos.* **115**, D14305 (2010).

43. T. R. Dallmann, R. A. Harley, T. W. Kirchstetter, Effects of diesel particle filter retrofits and accelerated fleet turnover on drayage truck emissions at the Port of Oakland. *Environ. Sci. Technol.* **45**, 10773–10779 (2011).

44. B. C. McDonald, T. R. Dallmann, E. W. Martin, R. A. Harley, Long-term trends in nitrogen oxide emissions from motor vehicles at national, state, and air basin scales. *J. Geophys. Res.* **117**, doi.org/10.1029/2012JD018304 (2012).

45. B. C. McDonald, A. H. Goldstein, R. A. Harley, Long-term trends in California mobile source emissions and ambient concentrations of black carbon and organic aerosol. *Environ. Sci. Technol.* **49**, 5178–5188 (2015).

46. C. V. Preble, T. R. Dallmann, N. M. Kreisberg, S. V. Hering, R. A. Harley, T. W. Kirchstetter, Effects of particle filters and selective catalytic reduction on heavy-duty diesel drayage truck emissions at the Port of Oakland. *Environ. Sci. Technol.* **49**, 8864–8871 (2015).

47. M. J. Haugen, G. A. Bishop, A. Thiruvengadam, D. K. Carder, Evaluation of heavy- and medium-duty on-road vehicle emissions in California's South Coast air basin. *Environ. Sci. Technol.* **52**, 13298–13305 (2018).

48. G. A. Bishop, Three decades of on-road mobile source emissions reductions in South Los Angeles. *J. Air Waste Manage. Assoc.* **69**, 967–976 (2019).

49. C. V. Preble, R. A. Harley, T. W. Kirchstetter, Control technology-driven changes to in-use heavy-duty diesel truck emissions of nitrogenous species and related environmental impacts. *Environ. Sci. Technol.* **53**, 14568–14576 (2019).

50. K. A. Yu, B. C. McDonald, R. A. Harley, Evaluation of nitrogen oxide emission inventories and trends for on-road gasoline and diesel vehicles. *Environ. Sci. Technol.* **55**, 6655–6664 (2021).

51. United States Environmental Protection Agency, Air pollutant emissions trends data (United States Environmental Protection Agency, 2023); www.epa.gov/air-emissions-inventories/air-pollutant-emissions-trends-data.

52. T. N. Skipper, A. S. Lawal, Y. Hu, A. G. Russell, Air quality impacts of electric vehicle adoption in California. *Atmos. Environ.* **294**, 119492 (2023).

53. U.S. Environmental Protection Agency, Official release of EMFAC2021 motor vehicle emission factor model for use in the State of California, Fed. Regist. 87, 68483–68489 (2022); www.govinfo.gov/content/pkg/FR-2022-11-15/pdf/2022-24790.pdf.

54. I. Mikati, A. F. Benson, T. J. Luben, J. D. Sacks, J. Richmond-Bryant, Disparities in distribution of particulate matter emission sources by race and poverty status. *Am. J. Public Health* **108**, 480–485 (2018).

55. C. Garcia, California State Assembly. An Act to Amend Sections 40920.6, 42400, and 42402 of, and to Add Sections 39607.1, 40920.8, 42411, 42705.5, and 44391.2 to, the Health and Safety Code, Relating to Nonvehicular Air Pollution. Assembly Bill No. 617. Nonvehicular Air Pollution: Criteria Air Pollutants and Toxic Air Contaminants (2017); https://leginfo.legislature.ca.gov/faces/billTextClient.xhtml?bill_id=201720180AB617.

56. K. De León, California State Senate. An Act to Add Sections 39711, 39713, 39715, 39721, and 39723 to the Health and Safety Code, Relating to Climate Change. Senate Bill No. 535. California Global Warming Solutions Act of 2006: Greenhouse Gas Reduction Fund (2012); https://leginfo.legislature.ca.gov/faces/billNavClient.xhtml?bill_id=201120120SB535.

57. California Office of Environmental Health Hazard Assessment, CalEnviroScreen 4.0 (California Office of Environmental Health Hazard Assessment, 2021); https://oehha.ca.gov/media/downloads/calenviroscreen/report/calenviroscreen40reportf2021.pdf.

58. A. van Donkelaar, M. S. Hammer, L. Bindle, M. Brauer, J. R. Brook, M. J. Garay, N. C. Hsu, O. V. Kalashnikova, R. A. Kahn, C. Lee, A. Lyapustin, A. M. Syer, R. V. Martin, Monthly global estimates of fine particulate matter and their uncertainty. *Environ. Sci. Technol.* **55**, 15287–15300 (2021).

59. S.-Y. Kim, M. Bechle, S. Hankey, L. Sheppard, A. A. Szpiro, J. D. Marshall, Concentrations of criteria pollutants in the contiguous U.S., 1979 – 2015: Role of prediction model parsimony in integrated empirical geographic regression. *PLOS ONE* **15**, e0228535 (2020).

60. California Air Resources Board, Air quality progress in California communities (California Air Resources Board, 2016); https://ww2.arb.ca.gov/sites/default/files/barcu/board/books/2016/062316/16-6-2pres.pdf.

61. D. H. Bennett, T. E. McKone, J. S. Evans, W. W. Nazaroff, M. D. Margni, O. Jolliet, K. R. Smith, Peer reviewed: Defining intake fraction. *Environ. Sci. Technol.* **36**, 206A–211A (2002).

62. J. D. Marshall, S. K. Teoh, W. W. Nazaroff, Intake fraction of nonreactive vehicle emissions in US urban areas. *Atmos. Environ.* **39**, 1363–1371 (2005).

63. J. S. Apte, E. Bombrun, J. D. Marshall, W. W. Nazaroff, Global intraurban intake fractions for primary air pollutants from vehicles and other distributed sources. *Environ. Sci. Technol.* **46**, 3415–3423 (2012).

64. B. Antonczak, T. M. Thompson, M. W. DePaola, G. Rowangould, 2020 near-roadway population census, traffic exposure and equity in the United States. *Transp. Res. D Transp. Environ.* **125**, 103965 (2023).

65. M. A. G. Demetillo, C. Harkins, B. C. McDonald, P. S. Chodrow, K. Sun, S. E. Pusede, Space-based observational constraints on NO_2 air pollution inequality from diesel traffic in major US cities. *Geophys. Res. Lett.* **48**, e2021GL094333 (2021).

66. V. Isakov, A. Venkatram, Resolving neighborhood scale in air toxics modeling: A case study in Wilmington, CA. *J. Air Waste Manag. Assoc.* **56**, 559–568 (2006).

67. L.-W. A. Chen, J. G. Watson, J. C. Chow, K. L. Magliano, Quantifying $PM_{2.5}$ source contributions for the San Joaquin Valley with multivariate receptor models. *Environ. Sci. Technol.* **41**, 2818–2826 (2007).

68. S. Hasheminassab, N. Daher, J. J. Schauer, C. Sioutas, Source apportionment and organic compound characterization of ambient ultrafine particulate matter (PM) in the Los Angeles Basin. *Atmos. Environ.* **79**, 529–539 (2013).

69. S. Hasheminassab, N. Daher, A. Saffari, D. Wang, B. D. Ostro, C. Sioutas, Spatial and temporal variability of sources of ambient fine particulate matter ($PM_{2.5}$) in California. *Atmos. Chem. Phys.* **14**, 12085–12097 (2014).

70. J. Hu, H. Zhang, S. Chen, Q. Ying, C. Wiedinmyer, F. Vandenberghe, M. J. Kleeman, Identifying $PM_{2.5}$ and $PM_{0.1}$ sources for epidemiological studies in California. *Environ. Sci. Technol.* **48**, 4980–4990 (2014).

71. C. Ng, B. Malig, S. Hasheminassab, C. Sioutas, R. Basu, K. Ebisu, Source apportionment of fine particulate matter and risk of term low birth weight in California: Exploring modification by region and maternal characteristics. *Sci. Total Environ.* **605–606**, 647–654 (2017).

72. R. Habre, M. Girguis, R. Urman, S. Fruin, F. Lurmann, M. Shafer, P. Gorski, M. Franklin, R. McConnell, E. Avol, F. Gilliland, Contribution of tailpipe and non-tailpipe traffic sources to quasi-ultrafine, fine, and coarse particulate matter in southern California. *J. Air Waste Manage. Assoc.* **71**, 209–230 (2021).

73. C. W. Tessum, J. D. Hill, J. D. Marshall, InMAP: A model for air pollution interventions. *PLOS ONE* **12**, e0176131 (2017).

74. D. L. Goldberg, M. Tao, G. H. Kerr, S. Ma, D. Q. Tong, A. M. Fiore, A. F. Dickens, Z. E. Adelman, S. C. Anenberg, Evaluating the spatial patterns of U.S. urban NOx emissions using TROPOMI NO_2. *Remote Sens. Environ.* **300**, 113917 (2024).

75. I. M. Dressel, M. A. G. Demetillo, L. M. Judd, S. J. Janz, K. P. Fields, K. Sun, A. M. Fiore, B. C. McDonald, S. E. Pusede, Daily satellite observations of nitrogen dioxide air pollution inequality in New York City, New York, and Newark, New Jersey: Evaluation and application. *Environ. Sci. Technol.* **56**, 15298–15311 (2022).

76. W. J. Requia, P. Koutrakis, Mapping distance-decay of premature mortality attributable to $PM_{2.5}$-related traffic congestion. *Environ. Pollut.* **243**, 9–16 (2018).

77. P. de Souza, S. Anenberg, C. Makarewicz, M. Shirgaokar, F. Duarte, C. Ratti, J. L. Durant, P. L. Kinney, D. Niemeier, Quantifying disparities in air pollution exposures across the united states using home and work addresses. *Environ. Sci. Technol.* **58**, 280–290 (2024).

78. D. Lekaki, M. Kastori, G. Papadimitriou, G. Mellios, D. Guizzardi, M. Muntean, M. Crippa, G. Oreggioni, L. Ntziachristos, Road transport emissions in EDGAR (Emissions Database for Global Atmospheric Research). *Atmos. Environ.* **324**, 120422 (2024).

79. S. Ma, D. Q. Tong, Neighborhood Emission Mapping Operation (NEMO): A 1-km anthropogenic emission dataset in the United States. *Sci. Data* **9**, 680 (2022).

80. H. Yang, X. Huang, D. M. Westervelt, L. Horowitz, W. Peng, Socio-demographic factors shaping the future global health burden from air pollution. *Nat. Sustain.* **6**, 58–68 (2023).

81. Q. Di, Y. Wang, A. Zanobetti, Y. Wang, P. Koutrakis, C. Choirat, F. Dominici, J. D. Schwartz, Air pollution and mortality in the medicare population. *N. Engl. J. Med.* **376**, 2513–2522 (2017).

82. Y. Ma, E. Zang, I. Opara, Y. Lu, H. M. Krumholz, K. Chen, Racial/ethnic disparities in $PM_{2.5}$-attributable cardiovascular mortality burden in the United States. *Nat. Hum. Behav.* **7**, 2074–2083 (2023).

83. K. P. Josey, S. W. Delaney, X. Wu, R. C. Nethery, P. DeSouza, D. Braun, F. Dominici, Air pollution and mortality at the intersection of race and social class. *N. Engl. J. Med.* **388**, 1396–1404 (2023).

84. F. J. Zimmerman, N. W. Anderson, Trends in health equity in the United States by race/ethnicity, sex, and income, 1993-2017. *JAMA Netw. Open* **2**, e196386 (2019).

85. P. Geldsetzer, D. Fridljand, M. V. Kiang, E. Bendavid, S. Heft-Neal, M. Burke, A. H. Thieme, T. Benmarhnia, Disparities in air pollution attributable mortality in the US population by race/ethnicity and sociodemographic factors. *Nat. Med.* 10.1038/s41591-024-03117-0 (2024).

86. W. H. McNeil, F. Tong, R. A. Harley, M. Auffhammer, C. D. Scown, Corridor-level impacts of battery-electric heavy-duty trucks and the effects of policy in the United States. *Environ. Sci. Technol.* **58**, 33–42 (2024).

87. Health Effects Institute, "Systematic review and meta-analysis of selected health effects of long-term exposure to traffic-related air pollution" (Spec. Rep. 23, Health Effects Institute, 2022); www.healtheffects.org/publication/systematic-review-and-meta-analysis-selected-health-effects-long-term-exposure-traffic.

88. D. C. S. Beddows, R. M. Harrison, PM_{10} and $PM_{2.5}$ emission factors for non-exhaust particles from road vehicles: Dependence upon vehicle mass and implications for battery electric vehicles. *Atmos. Environ.* **244**, 117886 (2021).

89. N. P. Nguyen, J. D. Marshall, Impact, efficiency, inequality, and injustice of urban air pollution: Variability by emission location. *Environ. Res. Lett.* **13**, 024002 (2018).

90. California Air Resources Board, Emissions spatial and temporal allocator model, Github Code Repository; https://github.com/mmb-carb/ESTA.

91. J. S. Manson, D. Van Riper, T. Kugler, S. Ruggles, IPUMS National Historical Geographic Information System, version 16.0, IPUMS (2021); http://doi.org/10.18128/D050.V16.0.

92. J. P. Allen, E. J. Turner, Patterns of population change in California 2000–2010, in *The California Geographer* (California Geographical Society, 2011), vol. 51, pp. 37–63; http://hdl.handle.net/10211.2/2814.
